# Repurposing of drug candidates against Epstein–Barr virus: Virtual screening, docking computations, molecular dynamics, and quantum mechanical study

**DOI:** 10.1371/journal.pone.0312100

**Published:** 2024-11-15

**Authors:** Mahmoud A. A. Ibrahim, Alaa M. A. Hassan, Eslam A. R. Mohamed, Gamal A. H. Mekhemer, Peter A. Sidhom, Mohamed A. El-Tayeb, Shahzeb Khan, Tamer Shoeib, Mahmoud E. S. Soliman, Alaa H. M. Abdelrahman

**Affiliations:** 1 Computational Chemistry Laboratory, Chemistry Department, Faculty of Science, Minia University, Minia, Egypt; 2 School of Health Sciences, University of KwaZulu-Natal, Westville Campus, Durban, South Africa; 3 Department of Pharmaceutical Chemistry, Faculty of Pharmacy, Tanta University, Tanta, Egypt; 4 Department of Botany and Microbiology, College of Science, King Saud University, Riyadh, Saudi Arabia; 5 Centre for Pharmaceutical Engineering Science, Faculty of Life Science, School of Pharmacy and Medical Sciences, University of Bradford, Bradford, United Kingdom; 6 Department of Chemistry, The American University in Cairo, New Cairo, Egypt; 7 Molecular Bio-Computation and Drug Design Laboratory, School of Health Sciences, University of KwaZulu-Natal, Westville Campus, Durban, South Africa; Nazarbayev University School of Medicine, PAKISTAN

## Abstract

Epstein–Barr virus (EBV) was the first tumor virus identified in humans, and it is mostly linked to lymphomas and cancers of epithelial cells. Nevertheless, there is no FDA-licensed drug feasible for this ubiquitous EBV viral contagion. EBNA1 (Epstein-Barr nuclear antigen 1) plays several roles in the replication and transcriptional of latent gene expression of the EBV, making it an attractive druggable target for the treatment of EBV-related malignancies. The present study targets EBV viral reactivation and upkeep by inhibiting EBNA1 utilizing a drug-repurposing strategy. To hunt novel EBNA1 inhibitors, a SuperDRUG2 database (> 4,600 pharmaceutical ingredients) was virtually screened utilizing docking computations. In accordance with the estimated docking scores, the most promising drug candidates then underwent MDS (molecular dynamics simulations). Besides, the MM-GBSA approach was applied to estimate the binding affinities between the identified drug candidates and EBNA1. On the basis of MM-GBSA//200 ns MDS, bezitramide (SD000308), glyburide (SD001170), glisentide (SD001159), and glimepiride (SD001156) unveiled greater binding affinities towards EBNA1 compared to KWG, a reference inhibitor, with Δ*G*_binding_ values of −44.3, −44.0, −41.7, −40.2, and −32.4 kcal/mol, respectively. Per-residue decomposition analysis demonstrated that LYS477, ASN519, and LYS586 significantly interacted with the identified drug candidates within the EBNA1 binding pocket. Post-dynamic analyses also demonstrated high constancy of the identified drug candidates in complex with EBNA1 throughout 200 ns MDS. Ultimately, electrostatic potential and frontier molecular orbitals analyses were performed to estimate the chemical reactivity of the identified EBNA1 inhibitors. Considering the current outcomes, this study would be an adequate linchpin for forthcoming research associated with the inhibition of EBNA1; however, experimental assays are required to inspect the efficiency of these candidates.

## Introduction

Epstein-Barr virus (EBV), or human herpesvirus 4, is a human lymphotropic herpesvirus widely distributed and linked to several malignancies [1]. EBV establishes a latent infection in B cells in more than 95% of the adult global population, which is considered a significant risk factor [2]. In addition to infecting B lymphocytes, EBV targets epithelial cells to produce infectious mononucleosis [3]. Once the EBV virus has successfully established latent infection in a cell, it may reactivate several times in the course of an individual’s life, leading to either lytic or abortive replication [4]. The virus’s lytic replication cycle generates offspring virions, which aid in the spread and upkeep of chronic infection [5]. Late-life reactivation of EBV is linked to several diseases, including lymphoproliferative disorders, NPC (nasopharyngeal carcinoma), and BL (Burkitt’s lymphoma) [6]. Intriguingly, clinical results have additionally indicated the function of EBV in many neurodegenerative diseases, such as Alzheimer’s illness [7–9]. EBV typically does not replicate in B cells but instead creates a latent infection marked by the restricted expression of a specific subset of viral latent genes [10]. Among the EBV genes, EBNA1 (Epstein-Barr Nuclear Antigen 1) plays a significant role in maintaining the latent viral genome in reproduced cells [11]. EBNA1 affects the EBV genome and host cells in various ways [12–14]. For instance, EBNA1 has a vital function in the upkeep and replication of the EBV genome through its sequence-specific attachment to the viral origin of replication [15]. Therefore, EBNA1 is considered a charming druggable target for treating EBV-related infection. A great deal of previous studies have been executed to identify potent EBNA1 inhibitors [16–18]. Nevertheless, no medication has yet been authorized as an EBNA1 inhibitor. Thus, the potentialities of other pharmaceutical compounds for EBNA1 still need to be inspected. Consequently, drug repurposing is therefore demanded as the fast track in the combat against EBV contagion.

**Fig 1:**
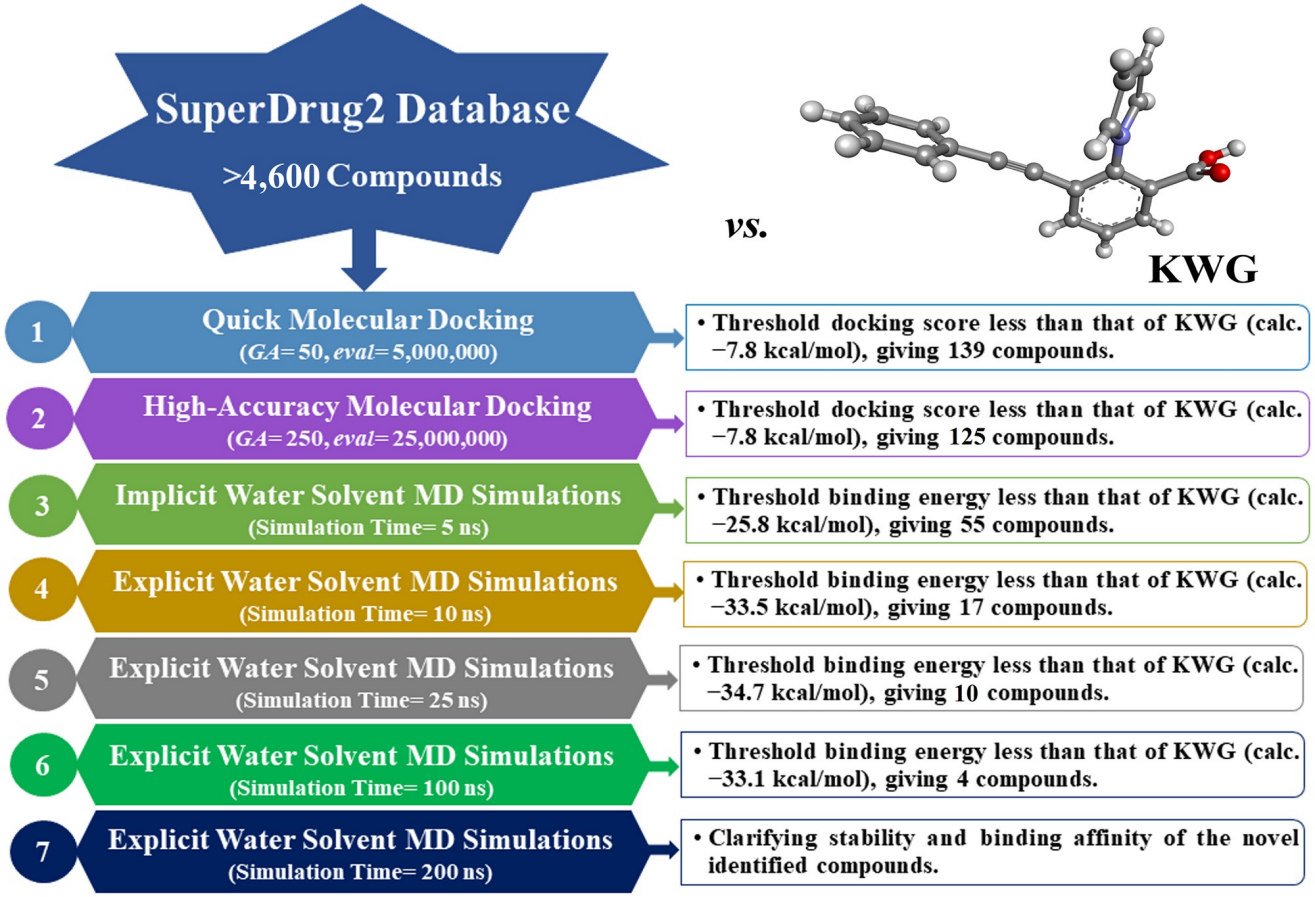
Schematic diagram of the utilized computational techniques for screening the SuperDRUG2 database against EBNA1.

In the current study, the SuperDRUG2 database containing more than 4,600 pharmaceutic ingredients was mined to identify putative EBNA1 inhibitors using *in-silico* techniques. On the basis of the docking predictions, the top-scoring compounds underwent MDS (molecular dynamics simulations), followed by binding affinity estimation using the MM-GBSA approach. The steadiness of the most potent drug candidates complexed with EBNA1 was additionally investigated over 200 ns MDS. To comprehend the reactive characteristics of the identified drug candidates in detail, DFT (density functional theory) computations were executed. Fig 1 depicts the workflow of the employed computational techniques to screen the SuperDRUG2 database. Conclusively, the current study’s findings illuminate the prospectivity of SuperDRUG2 candidates as EBNA1 inhibitors and, therefore, can become promising therapeutic agents for curtailing EBV infection. The major limitation of the current study is the lack of experimental validations of the identified EBNA1 inhibitors, and further *in-vivo* and *in-vitro* experiments need to be executed.

## Computational methodology

### Target preparation

The 3D structure of EBNA1 in complex with KWG (PDB code: 6NPP) was downloaded in the PDB format from the RCSB website [19]. All heteroatoms, small molecules, and ions were removed for the protein preparation. After that, the H++ web server was utilized to determine the protonation states of titratable amino acids, pursued by inserting H-atoms [20].

### SuperDRUG2 preparation

Prior to the virtual screening process, all compounds of the SuperDRUG2 database were downloaded in SDF format [21]. The duplicated drug candidates were eliminated according to their InChIKey (International Chemical Identifier) identifiers [22]. 3D conformers were generated from SDF utilizing Omega2 software [23, 24]. Moreover, the generated 3D conformers were minimized with the assistance of the MMFF94S force field within SZYBKI software [25, 26]. The Fixpka program inside the QUACPAC package was employed to investigate the ionization states of the inspected drug candidates [27]. For each drug candidate, the atomic charges were computed using the Gasteiger-Marsili method [28]. Prepared SuperDRUG2 compounds are accessible via www.compchem.net/ccdb.

### Molecular docking

AutoDock4.2.6 software was applied to execute all docking computations [29]. Following the docking protocol [30], the prepared EBNA1 protein was transformed into PDBQT format utilizing MGL1.5.7 tools. In the current study, two levels of docking computations were executed, namely quick and high-accuracy docking calculations, with *GA* (genetic algorithm) runs of 50 and 250, respectively. As well, *eval* (maximum number of energy evaluations) was adjusted to 5 and 250 million for quick and high-accuracy docking calculations, respectively. The grid was designed to encompass the whole binding pocket with dimensions of 50 × 50 × 50 Å^3^. The coordinates of the grid box center were positioned at *x* = −8.253, *y* = −34.005, and *z* = −15.597. The other parameters were left at their default settings. The docking pose with the lowest docking score was chosen as a representative binding mode.

### Molecular dynamics simulations (MDS)

The dynamic behavior of the top-ranking compounds complexed with EBNA1 was inspected using AMBER20 software [31]. More details about the parameters for running MDS are given elsewhere [32–35]. AMBER force field 14SB was adopted to characterize EBNA1 [36]. For the parameterization of the investigated drug candidates, GAFF2 (general AMBER force field) was employed [37]. In this work, implicit and explicit water solvents MDS were accomplished.

Within the framework of implicit water solvent MDS, the inspected drug candidates were minimized using the MMFF94S force field. The AM1-BCC approach was utilized to assign the inspected drug candidates atomic charges [38]. A cutoff was set to 999 Å for the nonbonded interactions. Non-periodic boundary conditions were also used. Additionally, to evaluate the solvation impact, the solvent model (implicit generalized born (igb) = 1) was used [39]. To minimize the docked drug candidates in the complex with EBNA1, 250 cycles of steepest descent and 250 cycles of conjugate gradient were carried out. After that, the minimized complexes were heated up to 310 K over 10 ps utilizing a Langevin thermostat. The heated complexes were then allowed to equilibrate for 50 ps. The equilibrated complexes were eventually subjected to a 5 ns production stage.

In the frame of explicit water solvent MDS, Gaussian09 software was used to optimize the studied inhibitors at the HF/6-31G* level of theory [40]. RESP (restrained electrostatic potential) approach was then employed to compute the atomic charges of the optimized inhibitors [41]. To solvate the systems, a truncated octahedron TIP3P water molecule with a margin of 1.2 nm was inserted [42]. Na^+^/Cl^−^ counterions were inserted to balance the drug-EBNA1 complexes’ charge. As well, 0.15 M NaCl was added in order to preserve an isosmotic state. Prior to the execution of MDS, minimization of the solvated complexes was carried out for 5,000 iterations. After that, the investigated systems were gradually heated up to 310 K over 50 ps. The equilibration phase for the inspected complexes was conducted over 10 ns under the NPT ensemble. Ultimately, the equilibrated complexes were subjected to production phases for 10, 25, 100, and 200 ns, and snapshots were recorded every 10 ps. MDS was conducted by the PMEMD.CUDA GPU implemented within AMBER 20 software. All interactions between EBNA1 and drug candidates were generated using BIOVIA Discovery Studio Visualizer [43].

### MM-GBSA binding energy

Evaluation of the binding affinities for drug-EBNA1 complexes was executed employing the MM-GBSA (molecular mechanics-generalized Born surface area) approach [44]. The following equation was used to compute binding energy (Δ*G*_binding_) according to the single-trajectory protocol:

$$\Delta G_{\text{binding}}=G_{\text{drug}-\text{EBNA}1}-\left(G_{\text{drug}}+G_{\text{EBNA}1}\right)$$ (1)

where the energy term (*G*) is calculated as:

$$G=E_{\text{vdW}}+G_{\text{GB}}+E_{\text{ele}}+\;G_{\text{SA}}$$ (2)

*E*_vdW_ points out van der Waals energy. *E*_ele_ implies electrostatic energy. *G*_SA_ and *G*_GB_ stand for the non-polar and polar participations of the desolvation energy, respectively. The *G*_GB_ was computed using the modified GB model (igb = 2) developed by Onufriev and colleagues [45]. The exterior and solute dielectric constants with values of 80 and 4, respectively, were utilized. Utilizing the LCPO method, the SASA (solvent-accessible surface area) was used to compute *G*_SA_ where *G*_SA_ = 0.0072 × SASA [46]. The entropic contribution was not considered because of its expensive computation cost [47, 48].

### Quantum mechanics computations

Quantum mechanical computations were performed on the identified EBNA1 inhibitors using Gaussian09 software [40]. Molecular structures of the EBNA1 inhibitors were collected from the MDS and subjected to geometrical optimization at M062X/6-311+G** level of theory. The optimized structures were then subjected to electrostatic potential (ESP) analysis. By means of ESP analysis, the maps of molecular electrostatic potential (MEP) were generated using an electron density envelope of 0.002 au [49].

From an electronic perspective, the optimized systems were examined using the frontier molecular orbitals (FMOs) theory. According to FMOs, the plots of the highest occupied and lowest unoccupied molecular orbitals (HOMO and LUMO, respectively) were generated. From the energy point of view, the *E*_HOMO_ and *E*_LUMO_ values were calculated for the drug candidates. Upon *E*_LUMO_ and *E*_HOMO_, the energy gap (*E*_gap_) value was computed as follows:

$$E_{\text{gap}}=E_{\text{LUMO}}-E_{\text{HOMO}}$$ (3)

For more electronic insights, global reactivity descriptors, including electron affinity (*EA*), ionization potential (*IP*), chemical potential (*μ*), global hardness (*η*), and global softness (*S*) were calculated as follows:

$$IP\approx -E_{\text{HOMO}}$$ (4)

$$EA\approx -E_{LUMO}$$ (5)

$$\eta =\frac{E_{\text{LUMO}}-E_{\text{HOMO}}}{2}$$ (6)

$$S=\;\frac{1}{\eta }$$ (7)

## Results and discussion

### Docking assessment

**Fig 2:**
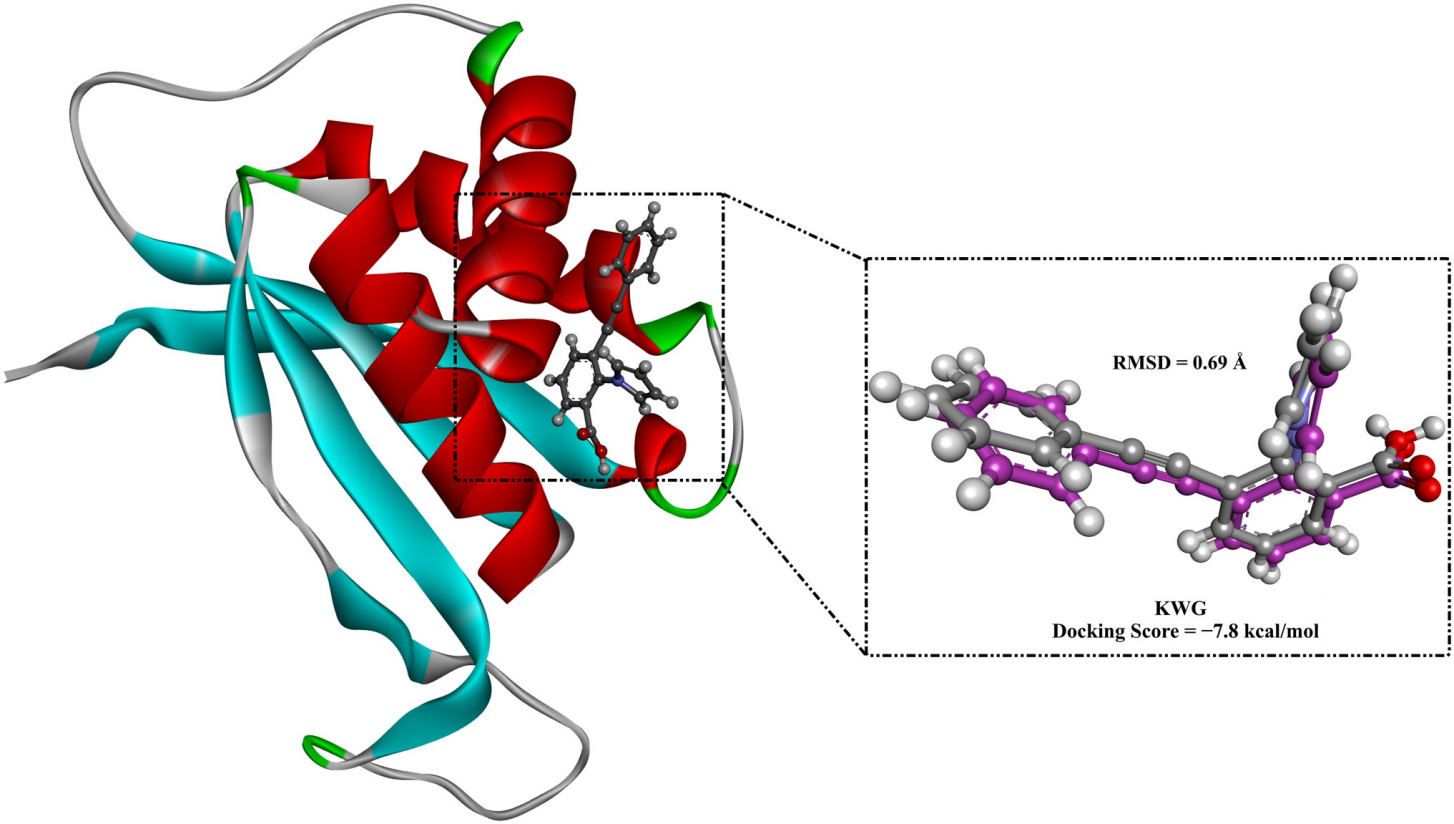
3D superimposition of the native structure (purple) and the anticipated docking pose (grey) of KWG against EBNA1.

To assess the employed docking protocol, re-docking of the co-crystallized KWG inhibitor was executed towards EBNA1. The docking outcomes displayed a good docking score of −7.8 kcal/mol. The calculated RMSD (root-mean-square deviation) between the native binding mode and the anticipated docking pose was found to be 0.69 Å, demonstrating the two poses almost entirely overlapped (Fig 2). These findings revealed that AutoDock4.2.6 software could accurately and successfully predict the correct binding mode of inhibitor inside the binding pocket of EBNA1. Therefore, the applied docking protocol was reliable for the virtual screening of the SuperDRUG2 database to identify potential EBNA1 inhibitors.

### Database screening

*In-silico* techniques have become an essential part of drug development, helping in the search for potent drug candidates [50, 51]. Over the past few decades, *in-silico* techniques have been developed and are broadly classified into structure-based or ligand-based strategies. Due to its low cost and efficiency, virtual screening (VS), a structure-based approach, is commonly used to predict the biological activity and binding modes of large chemical databases against a druggable target. Notably, compounds that have been optimized and virtually screened have shown promising potential in experimental tests in many instances [52]. Despite the relatively good explainability of *in-silico* techniques, their applicability is hindered by limited accuracy and the significant computational resources they require [52, 53]. The SuperDRUG2 database was mined for potent EBNA1 inhibitors using the validated docking approach. Initially, a quick docking protocol with *GA* = 50 and *eval* = 5,000,000 parameters was employed to virtually screen the SuperDRUG2 database against EBNA1. Upon the estimated docking scores, 139 drug candidates revealed lower docking scores in comparison with KWG (calc. −7.8 kcal/mol) towards EBNA1. These 139 drug candidates underwent more precise evaluations with high-accuracy docking parameters (i.e., *GA* = 250 and *eval* = 25,000,000). S1 Table summarizes the evaluated docking scores of these 139 drug candidates against EBNA1. As evident in S1 Table, 125 drug candidates manifested docking scores less than KWG (calc. −7.8 kcal/mol). Table 1 summarizes evaluated docking scores, and conventional H-bond of top potent drug candidates against EBNA1. The 3D and 2D molecular interactions of the anticipated binding modes for these drug candidates with EBNA1 are illustrated in S1 Fig. These drug candidates were chosen in accordance with the estimated binding affinities over 100 ns MDS. From S1 Fig, the molecular interactions displayed that most of the identified drug candidates demonstrated the same binding mode, establishing H-bonds with LYS477, LYS586, and ASN519 within the binding pocket of EBNA1. Besides, these drug candidates established various π-based and favorable hydrophobic interactions with the EBNA1 key residues.

**Table 1:** The computed quick and high-accuracy docking scores, and binding features for the most promising 10 drug candidates and KWG against EBNA1 ^a^.

Compound Name/Code	Docking Score (kcal/mol)		Conventional H-bond	
	Quick	High-Accuracy	Quick	High-Accuracy
**KWG**	–7.8	–7.8	ASN519 (1.98 Å),	ASN519 (1.96 Å)
**Bezitramide** (**SD000308)**	–9.5	–10.1	ASN519 (2.24 Å)	ASN519 (2.17 Å)
**Glyburide** **(SD001170)**	–9.1	–10.0	LYS477 (2.64 Å), LYS586 (2.43, 3.02 Å), THR590 (2.25 Å)	ASN480 (2.04, 2.20 Å), ASN519 (1.75 Å), LEU520 (2.42 Å), LYS586 (2.98 Å), THR590 (2.21 Å)
**Glisentide** **(SD001159)**	–9.6	–9.9	LYS477 (2.44 Å), ASN519 (1.88 Å), THR590 (2.20 Å)	ASN480 (2.02, 2.32 Å), ASN519 (1.84 Å), LEU520 (2.66 Å), THR590 (2.18 Å)
**Glimepiride** **(SD001156)**	–9.2	–9.8	LYS477 (2.68 Å), ASN519 (1.90 Å), LYS586 (2.34 Å), THR590 (1.91 Å)	LYS477 (2.67 Å), GLY484 (3.25 Å), LYS586 (2.24 Å), THR590 (1.82 Å)
**Ertapenem** **(SD000932)**	–9.0	–9.7	ASN480 (1.99 Å), ASN519 (2.02 Å), THR585 (2.71 Å),	LYS477 (3.08 Å), ASN480 (2.03 Å), ASN519 (2.03 Å), THR585 (1.97 Å), LYS586 (2.13 Å)
**Alatrofloxacin** **(SD000076)**	–8.4	–8.7	ASN480 (2.01 Å), GLY584 (2.10, 2.29 Å), THR585 (1.98 Å)	LYS477 (1.73 Å), ASN480 (2.02 Å), ASN519 (1.91, 2.62 Å), THR585 (2.01 Å)
**Delavirdine** **(SD000705)**	–8.3	–8.3	LYS477 (2.62 Å), ASN519 (2.18, 2.57 Å), THR585 (2.06 Å)	LYS477 (2.60 Å), ASN519 (2.20, 2.57 Å), THR585 (2.05 Å)
**Moxifloxacin** **(SD001650)**	–8.3	–8.3	LYS477 (2.77 Å), ASN480 (2.00, 1.66 Å), LYS586 (2.52 Å)	LYS477 (2.81, 3.06 Å), ASN480 (1.99 Å, 1.65 Å), LYS586 (2.51 Å)
**Clofazimine** **(SD000609)**	–8.2	–8.2	LYS586 (2.24 Å)	LYS586 (2.25 Å)
**Montelukast** **(SD001634)**	–8.0	–7.9	GLY484 (1.88 Å), LYS586 (1.80 Å)	GLY484 (1.87 Å), LYS586 (1.81 Å)

^a^ Data were sorted based on the high-accuracy docking scores.

^b^ Only H-bonds are demonstrated in Å.

**Fig 3:**
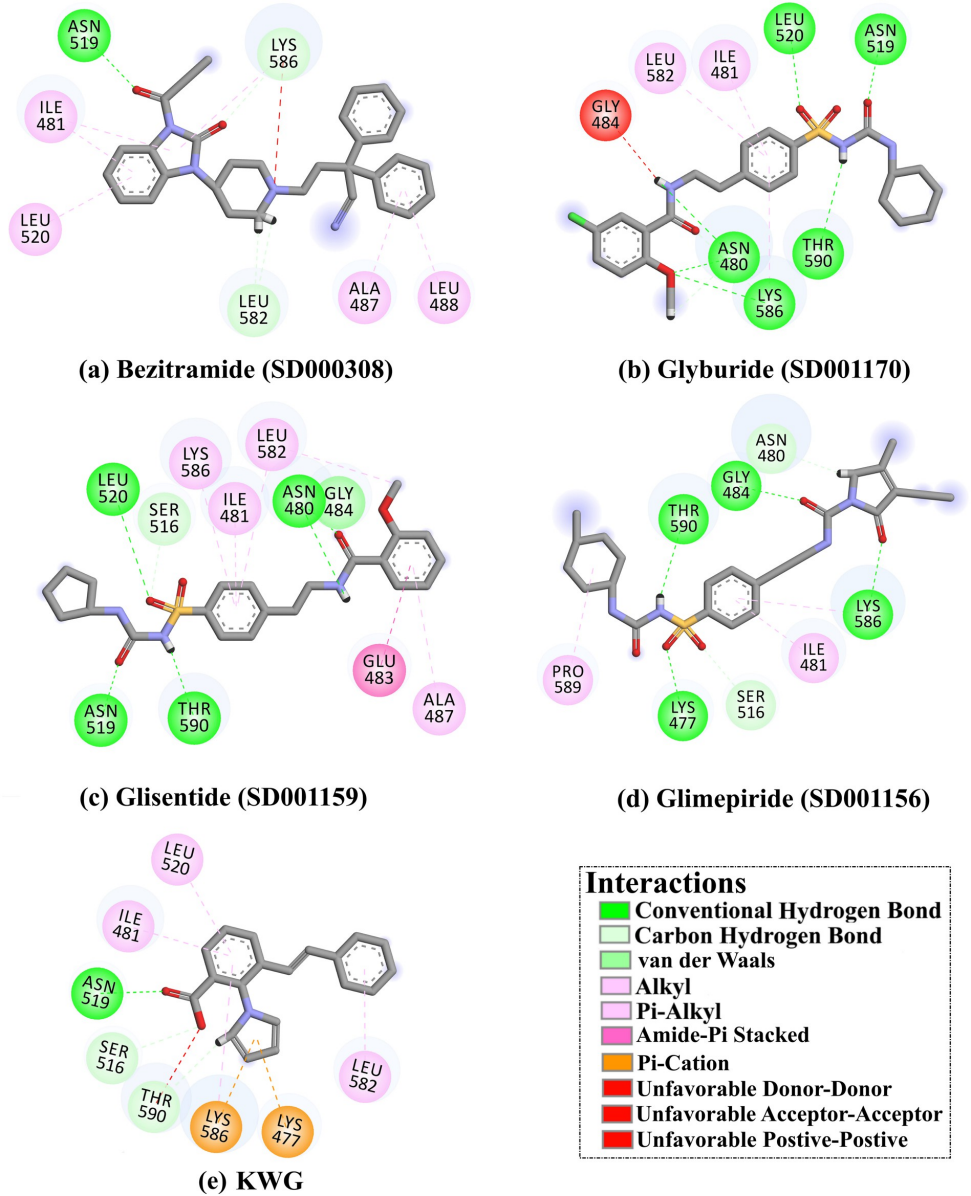
2D interaction diagram for (a) bezitramide (SD000308), (b) glyburide (SD001170), (c) glisentide (SD001159), (d) glimepiride (SD001156), and (e) KWG complexed with EBNA1.

Bezitramide (SD000308), a narcotic analgesic, exposed a superior docking score against EBNA1 with a value of −10.1 kcal/mol. Examining the binding mode of bezitramide inside the EBNA1 binding pocket demonstrated that the CO of the butan-2-one formed an H-bond with NH_2_ of ASN519 (2.17 Å). Besides, bezitramide exhibited three carbon-hydrogen bonds with LYS586 (2.31 Å) and LEU582 (2.82 and 2.59 Å) (Fig 3).

Glyburide (SD001170), which is used in treating non-insulin-dependent diabetes, demonstrated a good docking score of −10.0 kcal/mol. Analyzing the docking mode of glyburide revealed that OCH_3_ and *N*-methylformamide established two H-bonds with NH_2_ and CO of ASN480 with bond lengths of 2.20 and 2.04 Å, respectively. As well, SO_2_ demonstrated an H-bond with NH of LEU582 (2.42 Å). Besides, the 1,3-dimethylurea interacted with NH_2_ of ASN519 (1.75 Å) and OH of THR590 (2.21 Å). Ultimately, the OCH_3_ formed a carbon-hydrogen bond with ASN480 (2.74 Å).

Glisentide (SD001159) is prescribed orally to regulate blood sugar levels, disclosing a good docking score of −9.9 kcal/mol. Scrutinizing the binding mode of glisentide within the EBNA1 binding pocket unveiled that the 1,3-dimethylurea formed two H-bonds with OH of THR590 (2.21 Å) and NH_2_ of ASN519 (1.84 Å). Additionally, the *N*-methylformamide formed two H-bonds with CO and NH_2_ of ASN480 (2.32 and 2.02 Å). SO_2_ demonstrated an H-bond with NH of LEU582 (2.66 Å). Finally, glisentide displayed vdW interaction with GLY484 and amide π-stacking interaction with GLU483.

Glimepiride (SD001156), which is used to reduce blood sugar by stimulating insulin production by the pancreas, revealed a good docking score of −9.8 kcal/mol. According to its binding mode (Fig 3), SO_2_ of glimepiride exhibited an H-bond with NH_3_ of LYS477 (2.67 Å), and the 1,3-dimethylurea formed an H-bond with THR590 (1.82 Å). Besides, 1,3-dihydro-2H-pyrrol-2-one ring established an H-bond with LYS477 (2.24 Å). Furthermore, glimepiride demonstrated three carbon-hydrogen bonds with ASN480, GLY485, and SER516.

Finally, the positive control (KWG) unveiled a favorable docking score (calc. −7.8 kcal/mol). Inspecting the binding mode of the KWG within the EBNA1 binding pocket demonstrated that KWG formed only one H-bond with the NH_2_ of ASN519 (1.96 Å). Besides, KWG exhibited two pi-cation interactions with LYS586 and LYS477. As well, KWG formed two carbon-hydrogen bonds with THR590 and SER516.

### Molecular dynamics simulations (MDS)

**Fig 4:**
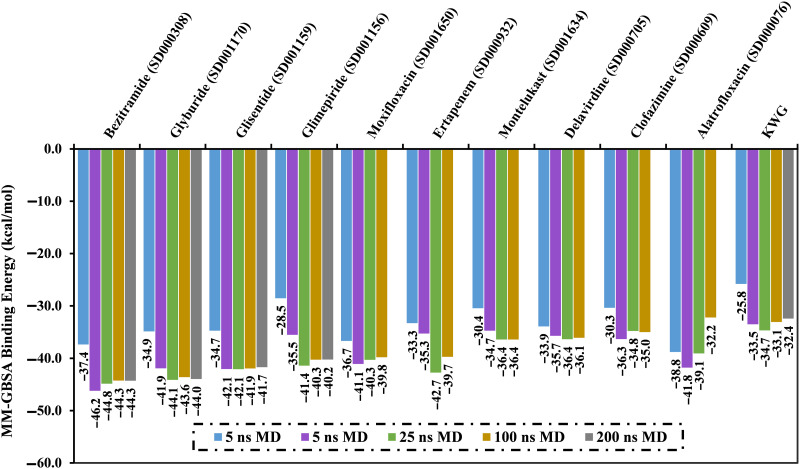
Computed binding energies for the KWG and the top potent drug candidates bound with EBNA1 over 5 ns MDS in implicit and 10 ns, 25 ns, 100 ns, and 200 ns MDS in explicit water solvents.

MDS is a significant theoretical tool for extensively inspecting the conformational changes and steadiness of the investigated receptors in the presence of inhibitors [54, 55]. Therefore, MDS was conducted for the most potent drug candidates with docking scores lower than −7.8 kcal/mol, accompanied by binding affinity estimation. The MDS was accomplished in implicit water solvent over 5 ns to reduce the computational time and expenses. The corresponding binding affinities were computed and are gathered in S2 Table. Upon data registered in S2 Table, 55 drug candidates unveiled lower binding energy in comparison with KWG (Δ*G*_binding_ = −25.8 kcal/mol). To attain more trusty binding affinities of drug candidates bound with EBNA1, these 55 drug candidates underwent 10 ns MDS in an explicit water solvent. Furthermore, the corresponding binding affinities were estimated and are enrolled in S3 Table. From S3 Table, 17 drug candidates demonstrated binding affinities greater than KWG (Δ*G*_binding_ = −33.1 kcal/mol). Accordingly, these 17 drug candidates were selected and underwent MDS for 25 ns, followed by binding affinity evaluations (S4 Table). As listed in S4 Table, 10 drug candidates displayed better binding affinities compared to KWG (Δ*G*_binding_ = −34.7 kcal/mol). These promising drug candidates were, therefore, submitted to a 100 ns MDS. The corresponding binding affinities were computed and are presented in Fig 4. Owing to the large number of drug candidates under investigation, a threshold value of −40.0 kcal/mol was chosen in order to shortlist the potential EBNA1 inhibitors. Based on the data in Fig 4, only four drug candidates—namely bezitramide, glyburide, glisentide, and glimepiride—unveiled binding energies less than −40.0 kcal/mol. For these four promising drug candidates complexed with EBNA1, MDS was extended to 200 ns, and the corresponding binding affinities were estimated in order to obtain more reliable results (Fig 4).

According to data in Fig 4, there is no discernible variation in the estimated MM-GBSA binding energies for the bezitramide-, glyburide-, glisentide-, and glimepiride-EBNA1 complexes throughout the 100 ns and 200 ns MDS. Based on the examined values in Fig 4, bezitramide, glyburide, glisentide, and glimepiride demonstrated lower binding energies against EBNA1 during 200 ns MDS, with Δ*G*_binding_ values of −44.3, −44.0, −41.7, and −40.2 kcal/mol, respectively, compared to KWG with Δ*G*_binding_ of −32.4 kcal/mol.

**Fig 5:**
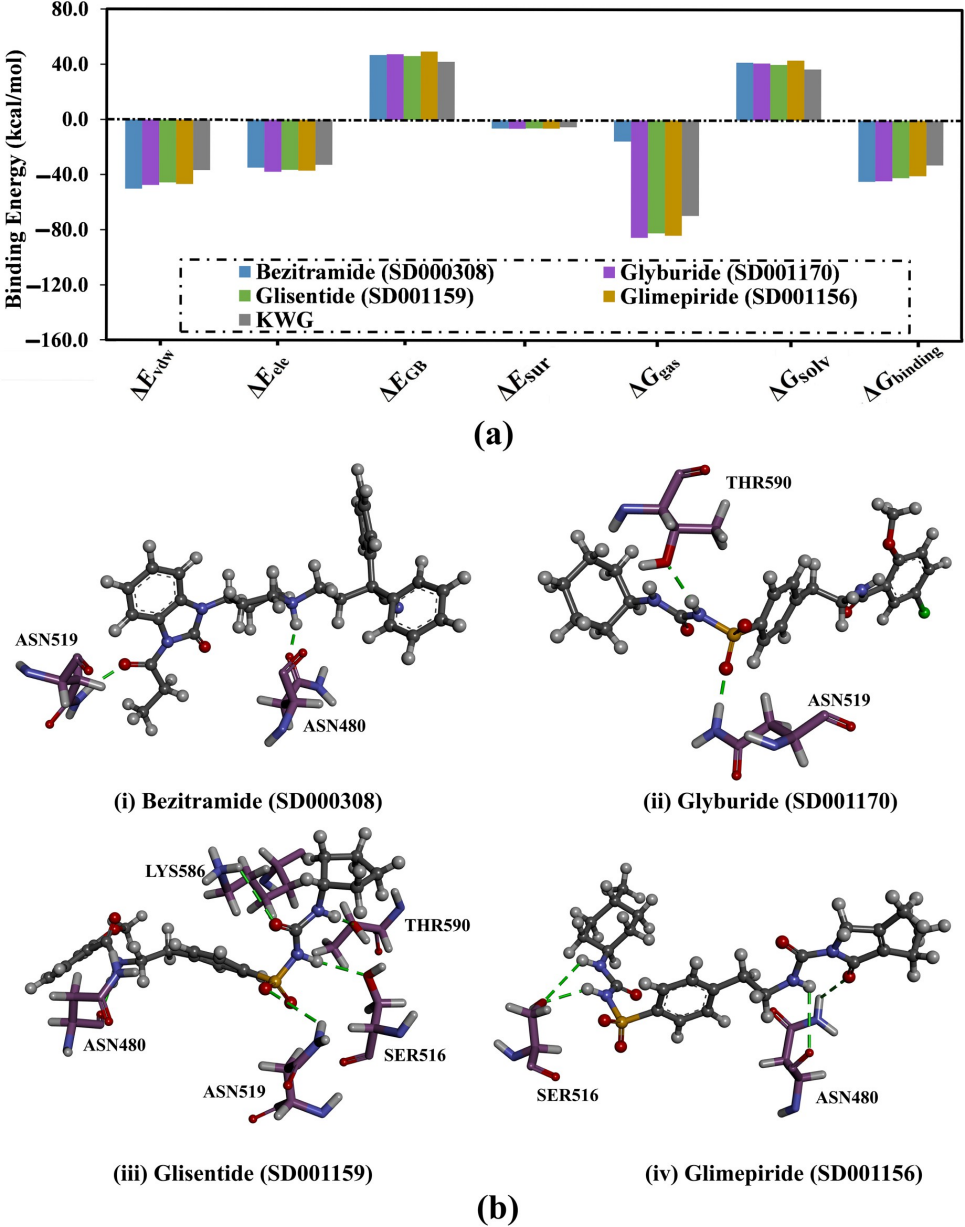
(a) Binding affinity components and (b) 3D representation of the average structure of (i) bezitramide (SD000308), (ii) glyburide (SD001170), (iii) glisentide (SD001159), and (iv) glimepiride (SD001156) bound with EBNA1 over 200 ns MDS.

To illuminate the nature of interactions between the identified drug candidates and EBNA1, the binding energy was decomposed, and the individual components of the binding energies were inspected (Fig 5a). As shown in Fig 5a, *E*_vdW_ is the dominant driving force of the binding affinities of bezitramide, glyburide, glisentide, and glimepiride, and KWG complexed with EBNA1 with values ranging from −36.7 to −50.2 kcal/mol. Additionally, *E*_ele_ displayed a favorable contribution to the binding energy of the identified drug candidates and KWG with EBNA1, with values in the range of −32.6 to −36.9 kcal/mol. The 3D molecular interactions of bezitramide-, glyburide-, glisentide-, and glimepiride-EBNA1 complexes based on the average structure throughout 200 ns MDS were investigated and are illustrated in Fig 5b. Examining the binding poses of the identified drug candidates within the EBNA1 binding pocket revealed that these candidates established new H-bonds with key amino acids inside the EBNA1 and preserved the original H-bond with ASN519. Interestingly, the new H-bonds were missing in the docking pose of the inspected drug candidates complexed with EBNA1, highlighting the significance of executing MDS. For explanatory purposes, bezitramide demonstrated a new H-bond with ASN480 (1.91 Å) and maintained its H-bond with ASN519 (1.86 Å).

**Fig 6:**
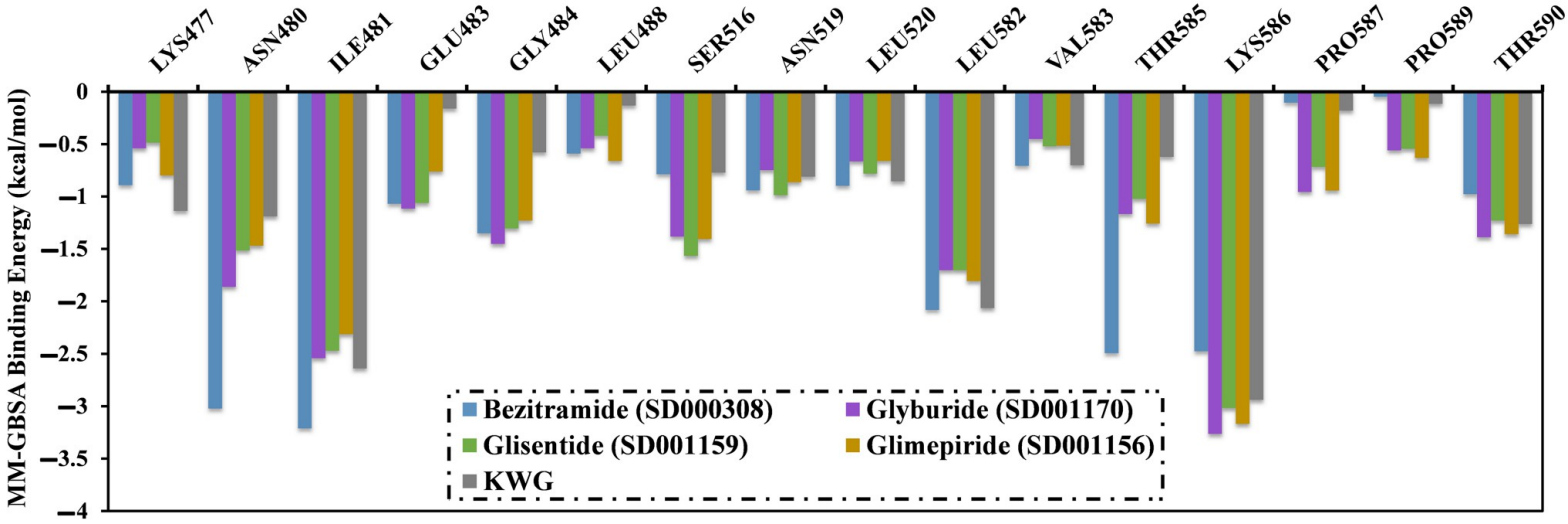
Illustration of per-residue energy decomposition analysis for bezitramide (SD000308)-, glyburide (SD001170)-, glisentide (SD001159)-, glimepiride (SD001156)-, and KWG-EBNA1 complexes over 200 ns MDS.

In addition to the computation of the total binding energy, per-residue energy decomposition analysis was executed to recognize the fundamental amino acids that participate considerably in the total binding energy (Fig 6). As shown in Fig 6, only residues with Δ*G*_binding_ < –0.5 kcal/mol were taken into account. LYS586 was found to have a significant contribution to Δ*G*_binding_, with values of –2.5, –3.3, –3.0, –3.2, and –2.9 kcal/mol for the bezitramide-, glyburide-, glisentide-, glimepiride-, and KWG-EBNA1 complexes, respectively.

### Post-MD analyses

Post-MD analyses should be conducted to inspect the steadiness of receptor-inhibitor complexes over MDS [56–58]. After conducting 200 ns MDS for the identified drug candidates complexed with EBNA1, post-MD analyses were executed to observe the structural and energetical changes. These analyses involving binding energy analysis, RMSD and RMSF (root-mean-square deviation and fluctuation), CoM (center of mass) distance, H-bond analysis, and Rg (radius of gyration) were gauged as a function of time.

#### Binding energy analysis

**Fig 7:**
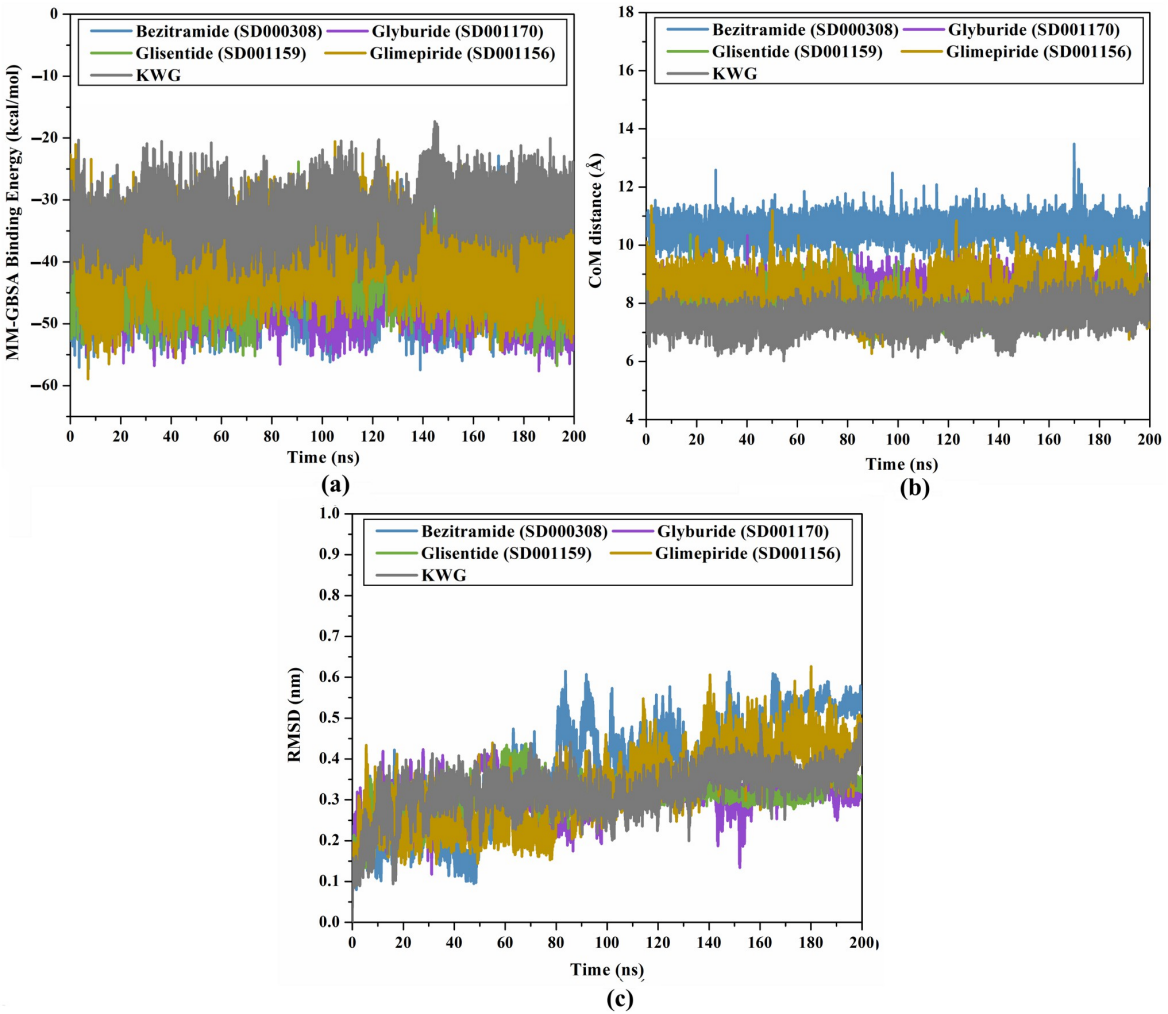
(a) Binding energy analysis, (b) CoM distance, and (c) RMSD of bezitramide (SD000308) (blue), glyburide (SD001170) (mauve), glisentide (SD001159) (green), glimepiride (SD001156) (dark yellow), and KWG (grey) towards EBNA1 throughout 200 ns MDS.

For inspecting the energetical analysis of bezitramide-, glyburide-, glisentide-, glimepiride-, and KWG-EBNA1 complexes throughout 200 ns MDS, the binding energy per frame was estimated and is plotted in Fig 7a. As demonstrated in Fig 7a, the general constancy was observed for bezitramide-, glyburide-, glisentide-, glimepiride-, and KWG-EBNA1 complexes with average Δ*G*_binding_ values of −44.3, −44.0, −41.7, −40.2, and −32.4 kcal/mol, respectively. This analysis shows that the investigated complexes maintained their stability over 200 ns MDS.

#### CoM distance

To better understand the stability of drug-EBNA1 complexes over the 200 ns MDS, the CoM of each drug candidate and ASN519 was measured (Fig 7b). As displayed in Fig 7b, the CoM distance was steady for bezitramide, glyburide, glisentide, glimepiride, and KWG in complex with EBNA1 with average values of 10.5, 8.4, 8.1, 8.5, and 7.5 Å, respectively. The most important finding from CoM analyses was that the identified drug candidates were bound rigidly to EBNA1.

#### RMSD analysis

To track the conformational variations in the drug-EBNA1 complexes during MDS, the RMSD for the backbone atoms of the bezitramide, glyburide, glisentide, glimepiride, and KWG complexed with EBNA1 concerning the initial structure was computed for each MD frame. Fig 7c shows RMSD *vs*. time for the investigated drug candidates over 200 ns MDS. Looking at Fig 7c, it is apparent that the RMSD values for the inspected drug candidates complexed with EBNA1 were less than 0.5 nm throughout 200 ns MDS. These findings indicated no significant variations in the inspected complexes, and the investigated drug candidates stayed in the EBNA1 binding pocket over the 200 ns MDS.

#### H-bond analysis

**Fig 8:**
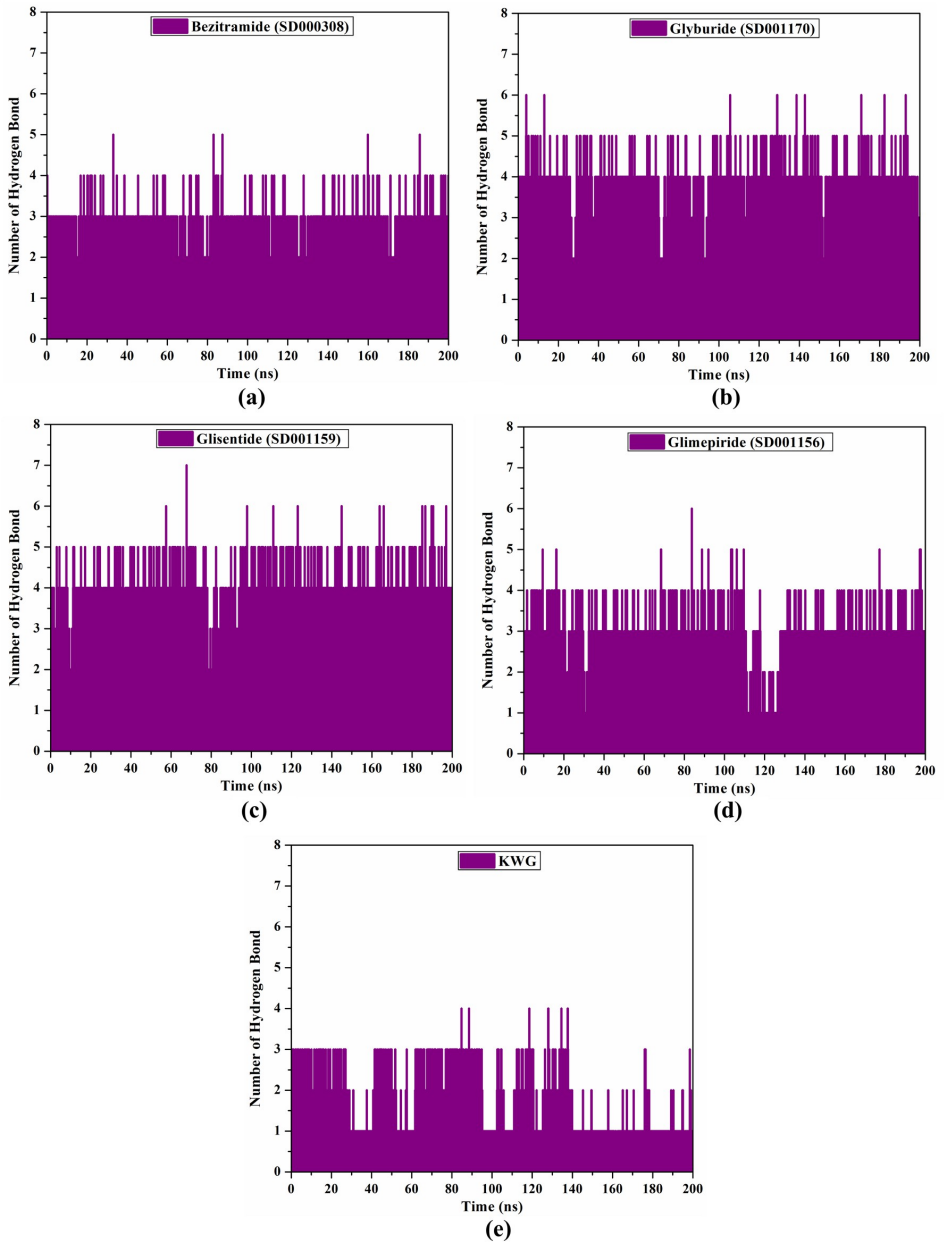
Number of H-bonds for (a) bezitramide (SD000308), (b) glyburide (SD001170), (c) glisentide (SD001159), (d) glimepiride (SD001156), and (e) KWG complexed with EBNA1 over 200 ns MDS.

Since the formation of stable receptor-inhibitor complexes depends on H-bonds, the number of intermolecular H-bonds for each drug candidate complexed with EBNA1 over 200 ns MDS was also examined. Fig 8 illustrates the number of H-bonds for bezitramide, glyburide, glisentide, glimepiride, and KWG complexed with EBNA1 over 200 ns MDS. As obvious in Fig 8, the number of H-bonds were 3, 4, 4, 3, and 2 for bezitramide, glyburide, glisentide, glimepiride, and KWG complexed with EBNA1 over 200 ns MDS, respectively. This data suggests that these drug candidates stably interacted with the EBNA1 active site over 200 ns MDS.

#### RMSF analysis

**Fig 9:**
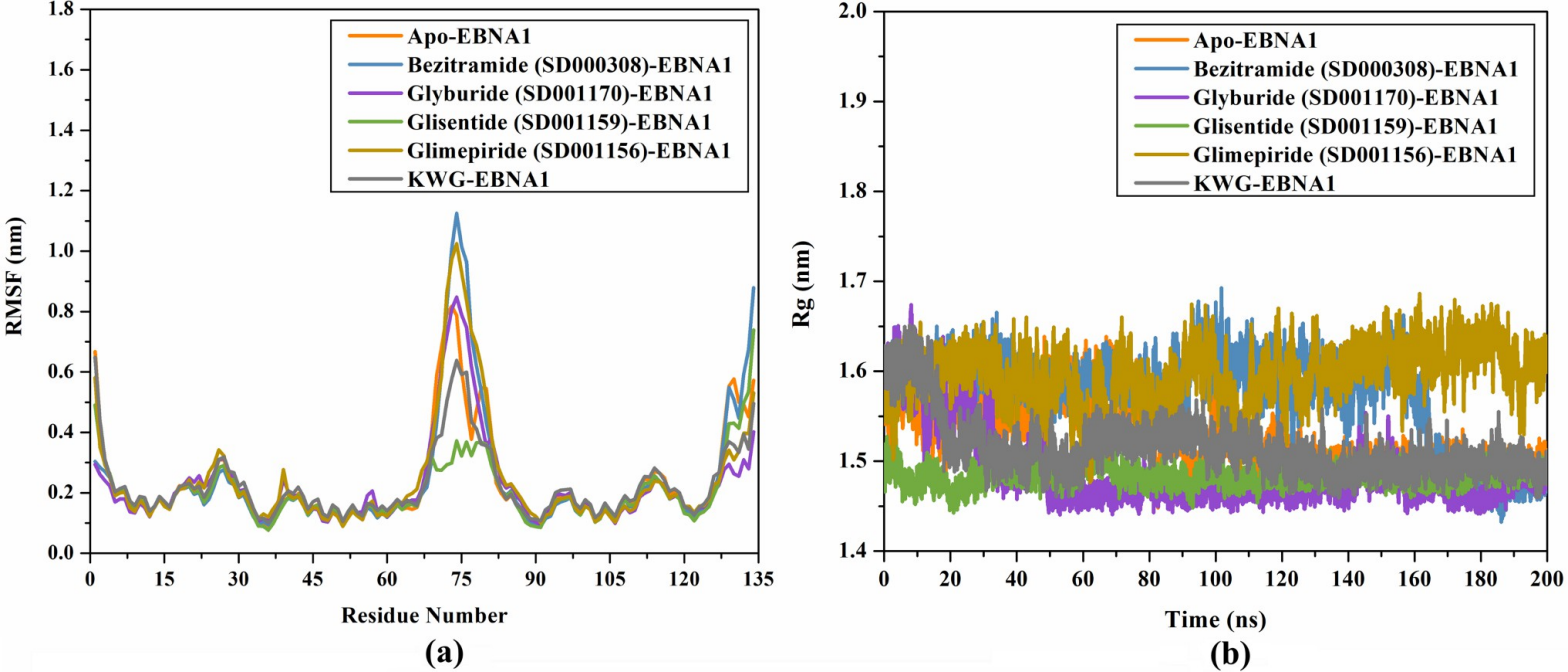
(a) RMSF and (b) Rg of apo-EBNA1 (orange), bezitramide (SD000308)-EBNA1 (blue), glyburide (SD001170)-EBNA1 (mauve), glisentide (SD001159)-EBNA1 (green), glimepiride (SD001156)-EBNA1 (dark yellow), and KWG-EBNA1 (grey) over 200 ns MDS.

Measuring the RMSF is also a valuable analysis for determining the structural steadiness of the receptor-ligand complex over MDS. The RMSF analysis is identical to the RMSD, except that individual residue flexibility is used. According to RMSF, the degree of constancy increases with decreasing coordinate fluctuation. Fig 9a displays the RMSF of C_*α*_ of apo-EBNA1, bezitramide-, glyburide-, glisentide-, glimepiride-, and KWG-EBNA1 complexes over 200 ns MDS. From Fig 9a, the amino acids were observed to steady in bezitramide-, glyburide-, glisentide-, glimepiride-, and KWG-EBNA1 complexes over 200 ns MDS.

#### Rg analysis

The Rg analysis was performed to estimate the general compactness and steadiness of the investigated inhibitor-receptor complex over a simulation time. The Rg plot for apo-EBNA1, bezitramide-, glyburide-, glisentide-, glimepiride-, and KWG-EBNA1 complexes over 200 ns MDS is shown in Fig 9b. The Rg values for apo-EBNA1, bezitramide-, glyburide-, glisentide-, glimepiride-, and KWG-EBNA1 complexes were 1.50, 1.65, 1.52, 1.51, and 1.53 nm, respectively (Fig 9b). Conclusively, the complexation of protein with the identified drug candidates increases the compactness and rigidity of the EBNA1 structure, which in turn leads to increased general steadiness (Fig 9b).

#### Quantum mechanical calculations

**Fig 10:**
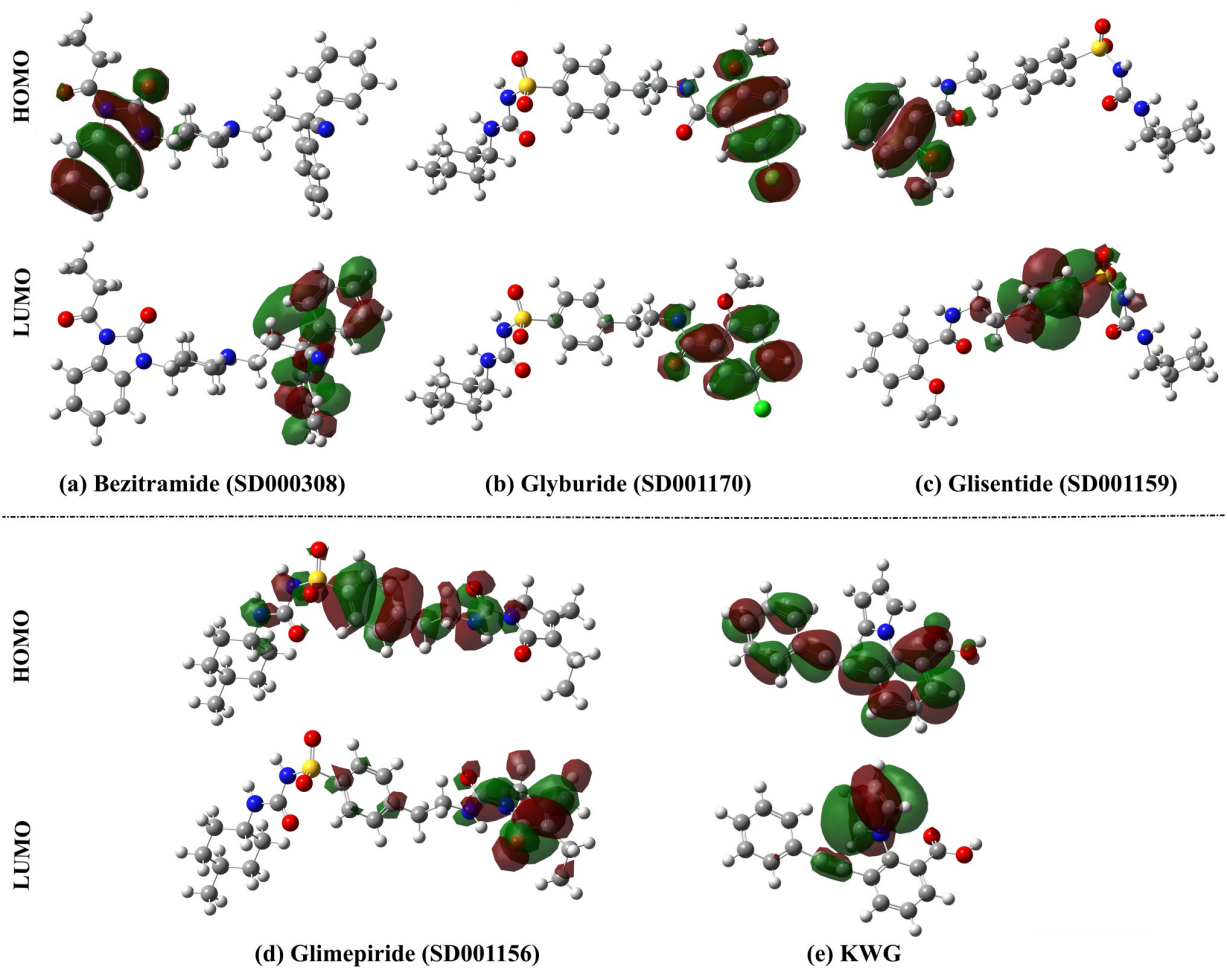
MEP maps of the final trajectory of (a) bezitramide (SD000308), (b) glyburide (SD001170), (c) glisentide (SD001159), (d) glimepiride (SD001156), and (e) KWG.

Electrostatic potential (ESP) analysis is an efficient tool for illustrating the charge distribution over the molecular structure [54]. Upon the ESP analysis, MEP maps were generated and plotted for the last trajectory of drug candidates and KWG extracted from MDS (Fig 10).

According to Fig 10, various electrophilic (i.e., blue-colored regions) and nucleophilic (i.e., red-colored regions) sites were observed over the studied drug candidates and KWG. Notably, the negative potentials were found over O and N atoms, while positive potentials were located over H atoms. By analyzing MEP maps of the investigated compounds, these compounds demonstrated the capacity to exhibit H-bonds with the key residues inside the EBNA1 active site.

**Fig 11:**
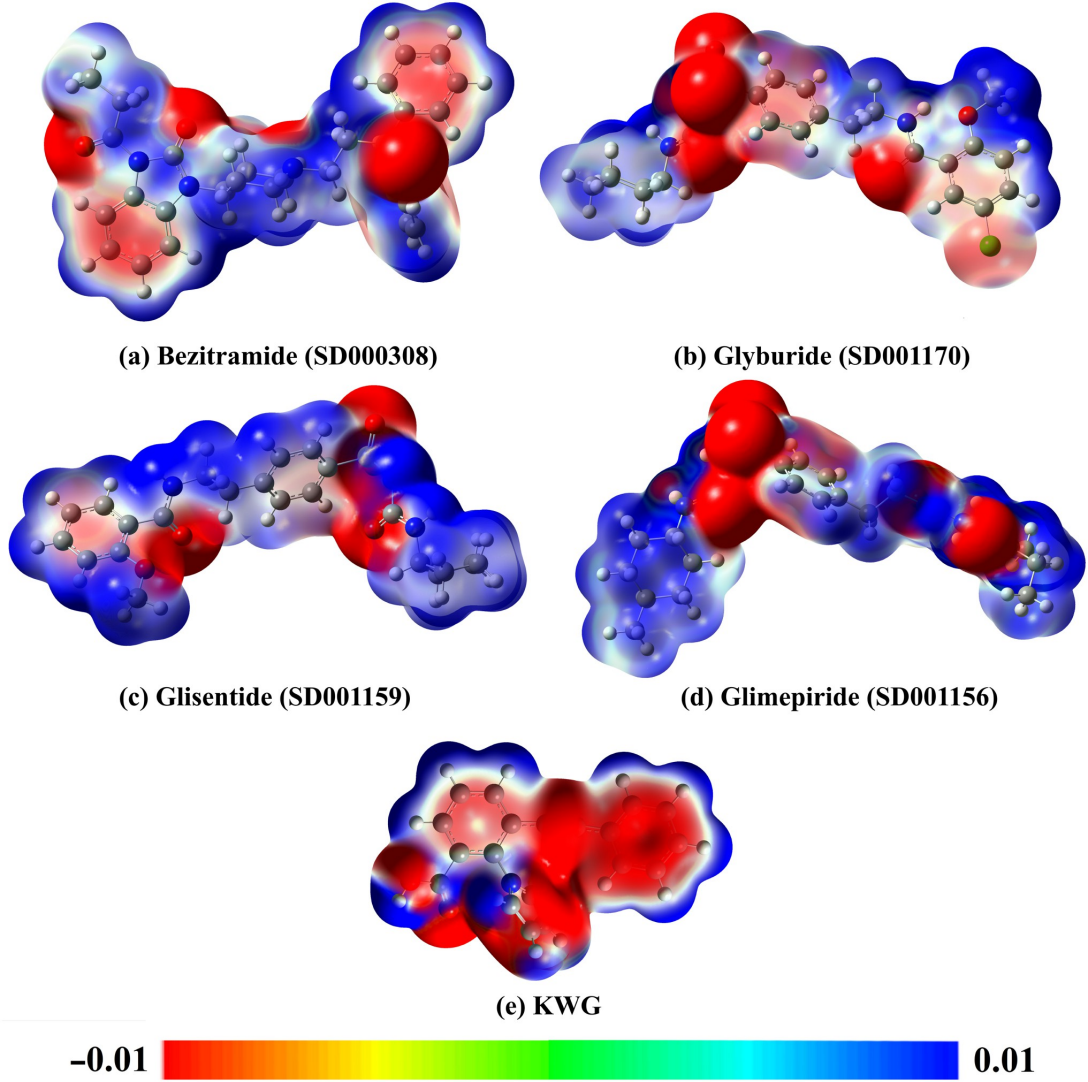
HOMO and LUMO plots of (a) bezitramide (SD000308), (b) glyburide (SD001170), (c) glisentide (SD001159), (d) glimepiride (SD001156), and (e) KWG.

Frontier Molecular Orbitals (FMOs) are primarily utilized to predict various molecular characteristics, including molecular interactions, reactivity, charge transfer, optical properties, and bioactivity [59–62]. In terms of electronic aspects, FMOs theory was applied to the inspected drug candidates and KWG as prospective EBNA1 inhibitors. Within FMOs analysis, HOMO and LUMO plots were generated and mapped for the inspected drug candidates and KWG (Fig 11). Table 2 collects the *E*_HOMO_, *E*_LUMO_, and *E*_gap_ values of the optimized structures of the most promising drug candidates and KWG. As shown in Fig 11, HOMO levels were mainly found around O and N atoms, whereas LUMO levels were observed over H atoms of the investigated drug candidates and KWG.

**Table 2:** *E*_HOMO_, *E*_LUMO_, and *E*_gap_ values of the most promising drug candidates and KWG.

Compound Name/Code	*E*_HOMO_ (eV)	*E*_LUMO_ (eV)	*E*_gap_ (eV)
**KWG**	–7.17	–1.15	6.03
**Bezitramide (SD000308)**	–7.49	–0.14	7.35
**Glyburide (SD001170)**	–8.03	–0.72	7.31
**Glisentide (SD001159)**	–7.85	–0.36	7.50
**Glimepiride (SD001156)**	–8.39	–0.46	7.92

According to data in Table 2, the identified drug candidates and KWG revealed low *E*_gap_ values ranging from 6.03 to 7.92 eV, illustrating their high chemical reactivity. Looking at Table 2, it is apparent that the chemical reactivity of the identified drug candidates and KWG can be sorted upon their *E*_gap_ in the following order: Glimepiride < glisentide < bezitramide < glyburide < KWG.

Based on the calculated values of *E*_HOMO_ and *E*_LUMO_, different global indices parameters were estimated and gathered in Table 3. From the data in Table 3, the *EA* and *IP* values demonstrated a similar manner to that of the *E*_LUMO_ and *E*_HOMO_ values, respectively. This resemblance may be attributed to the fact that the *EA* and *IP* values are mostly computed based on the *E*_LUMO_ and *E*_HOMO_ values, respectively. The chemical stability and reactivity of the optimized drug candidates and KWG were demonstrated by their hardness (*η*) and softness (*S*), respectively. The hardness values of the inspected drug candidates decreased in the following order: Glimepiride > glisentide > bezitramide > glyburide > KWG. As evident in Table 3, the degree of softness of the inspected drug candidates increased upon the following pattern: Glimepiride < glisentide < bezitramide < glyburide < KWG.

**Table 3:** Global indices reactivity parameters of the inspected drug candidates and KWG.

Compound Name/Code	*IP* (eV)	*EA* (eV)	*η* (eV)	*S* (eV−^1^)
**KWG**	7.17	1.14	3.01	0.33
**Bezitramide (SD000308)**	7.49	0.14	3.67	0.27
**Glyburide (SD001170)**	8.03	0.72	3.65	0.27
**Glisentide (SD001159)**	7.85	0.36	3.75	0.27
**Glimepiride (SD001156)**	8.39	0.46	3.96	0.25

## Conclusion

EBV is responsible for a considerable number of fatalities in African and Asian people, and its latent infection is linked to multiple cancers in humans. EBNA1 is an attractive druggable target for identifying therapeutic drug candidates to combat EBV infection due to its crucial role in viral replication. In the current study, the SuperDRUG2 database, including > 4,600 drug candidates, was screened to identify putative EBNA1 inhibitors using *in-silico* techniques. Upon docking predictions and MDS accompanied by binding energy estimations, bezitramide, glyburide, glisentide, and glimepiride demonstrated promising affinities with Δ*G*_binding_ less than −40.0 kcal/mol towards EBNA1. The steadiness of the investigated drug candidates bound with EBNA1 was verified utilizing post-MD analyses through 200 ns. Further, FMO theory and MEP analysis were also executed, and their results supported the outcomes obtained from docking predictions and MDS. The current study demonstrated that bezitramide, glyburide, glisentide, and glimepiride are promising EBNA1 inhibitors. Despite the promising findings of the current study, one limitation is the lack of experimental validation. However, the current findings would help speed up the discovery process of EBNA1 inhibitors.

## Supporting information

S1 Fig The 3D and 2D molecular interactions of the anticipated binding modes for the top 10 candidates with EBNA1. (DOCX)

S1 Table The anticipated quick and high-accuracy docking scores (in kcal/mol) for the top 139 SuperDrug2 compounds and KWG towards EBNA1. (DOCX)

S2 Table The anticipated quick and high-accuracy docking scores and MM-GBSA binding energies (in kcal/mol) over 5 ns implicit MD simulation for the top 125 SuperDRUG2 compounds and KWG towards EBNA1. (DOCX)

S3 Table The anticipated quick and high-accuracy docking scores and MM-GBSA binding energies (in kcal/mol) over 5 ns implicit and 5 ns explicit MDS for the top 55 SuperDRUG2 compounds and KWG towards EBNA1. (DOCX)

S4 Table The anticipated quick and high-accuracy docking scores and MM-GBSA binding energies (in kcal/mol) over 5 ns implicit and 5 ns and 25 ns explicit MDS for the top 17 SuperDRUG2 compounds and KWG towards EBNA1. (DOCX)

S1 Graphical abstract (TIF)
