# Supplementary material for: Repurposing of drug candidates against Epstein–Barr virus: Virtual screening, docking computations, molecular dynamics, and quantum mechanical study
Source: PLoS One. 2024 Nov 15;19(11):e0312100. doi: 10.1371/journal.pone.0312100 (PMC11567563; doi:10.1371/journal.pone.0312100)
Supplement: S1 Fig — (DOCX) [file pone.0312100.s001.docx]

**
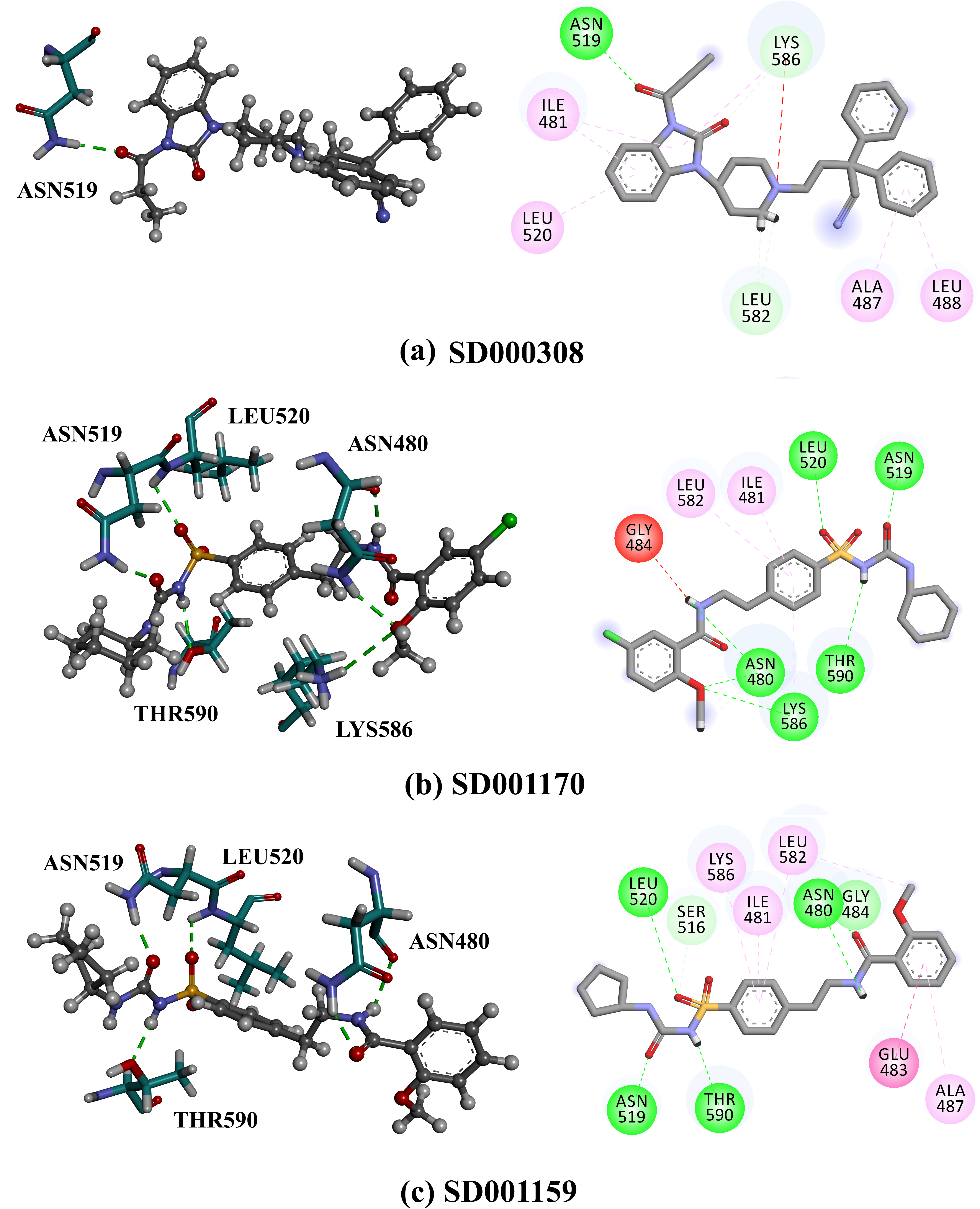
**

**S1 Fig.** The 3D and 2D molecular interactions of the anticipated binding modes for the top 10 candidates with EBNA1.


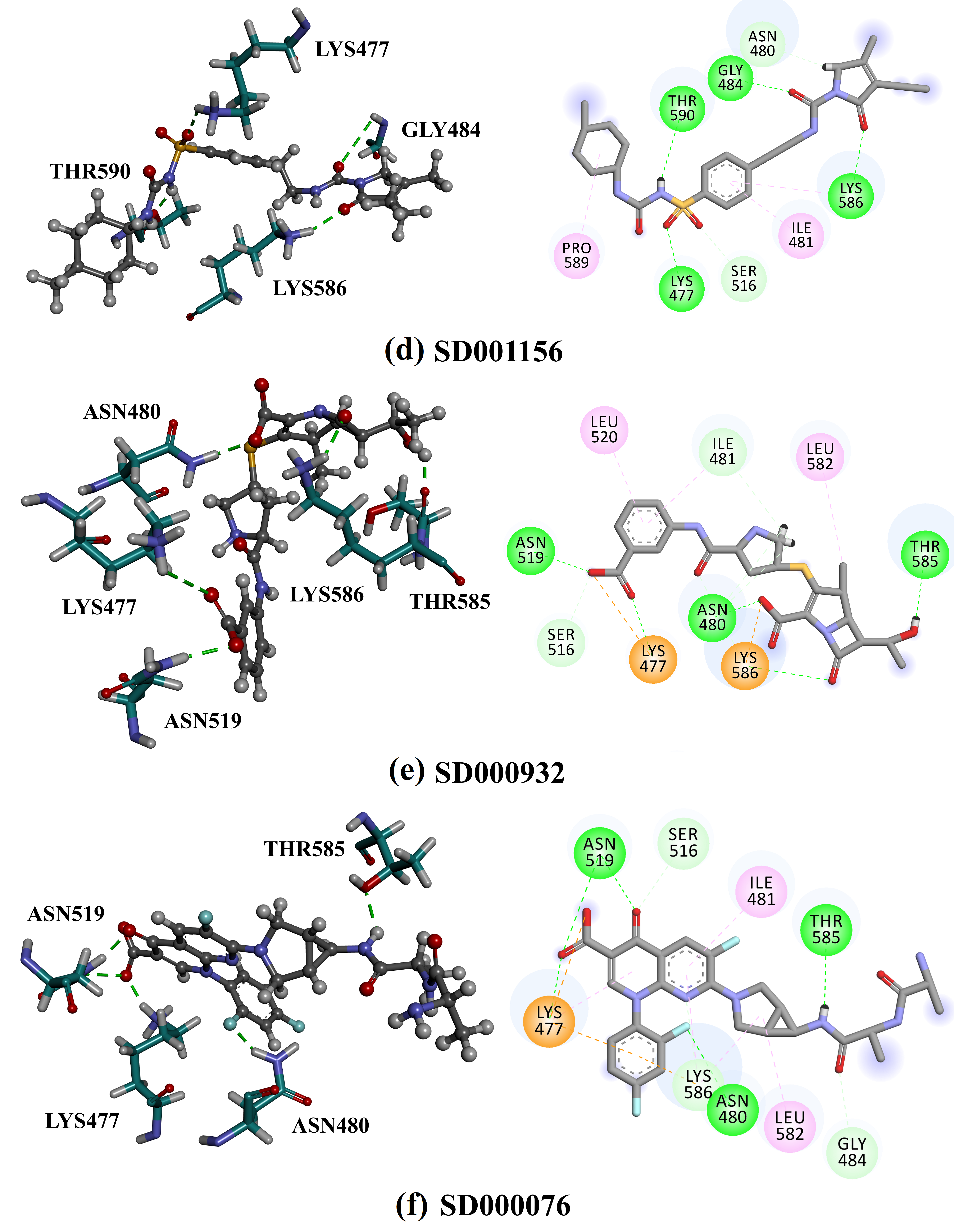


**S1 Fig.** *Continued.*

**
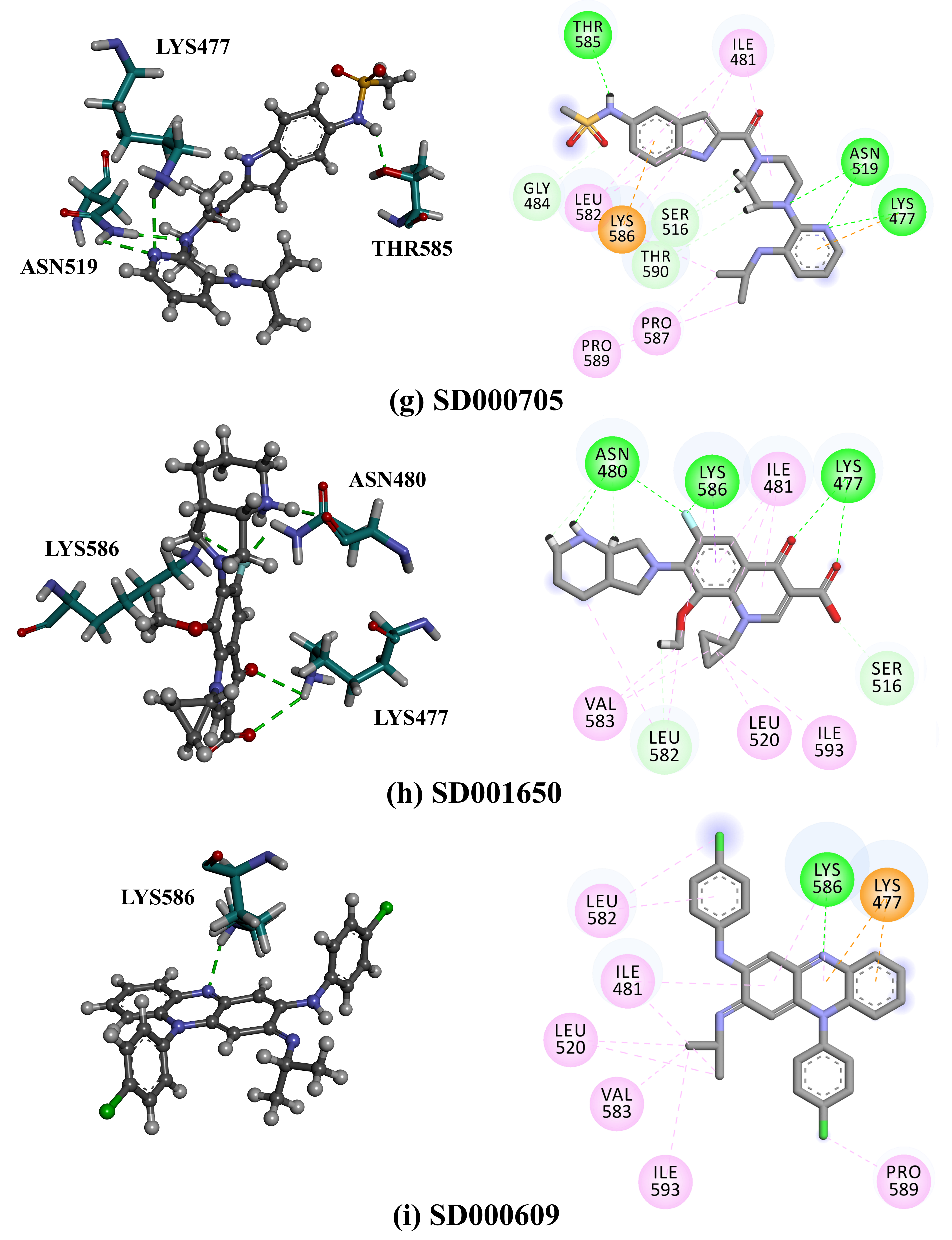
**

**S1 Fig.** *Continued.*

*
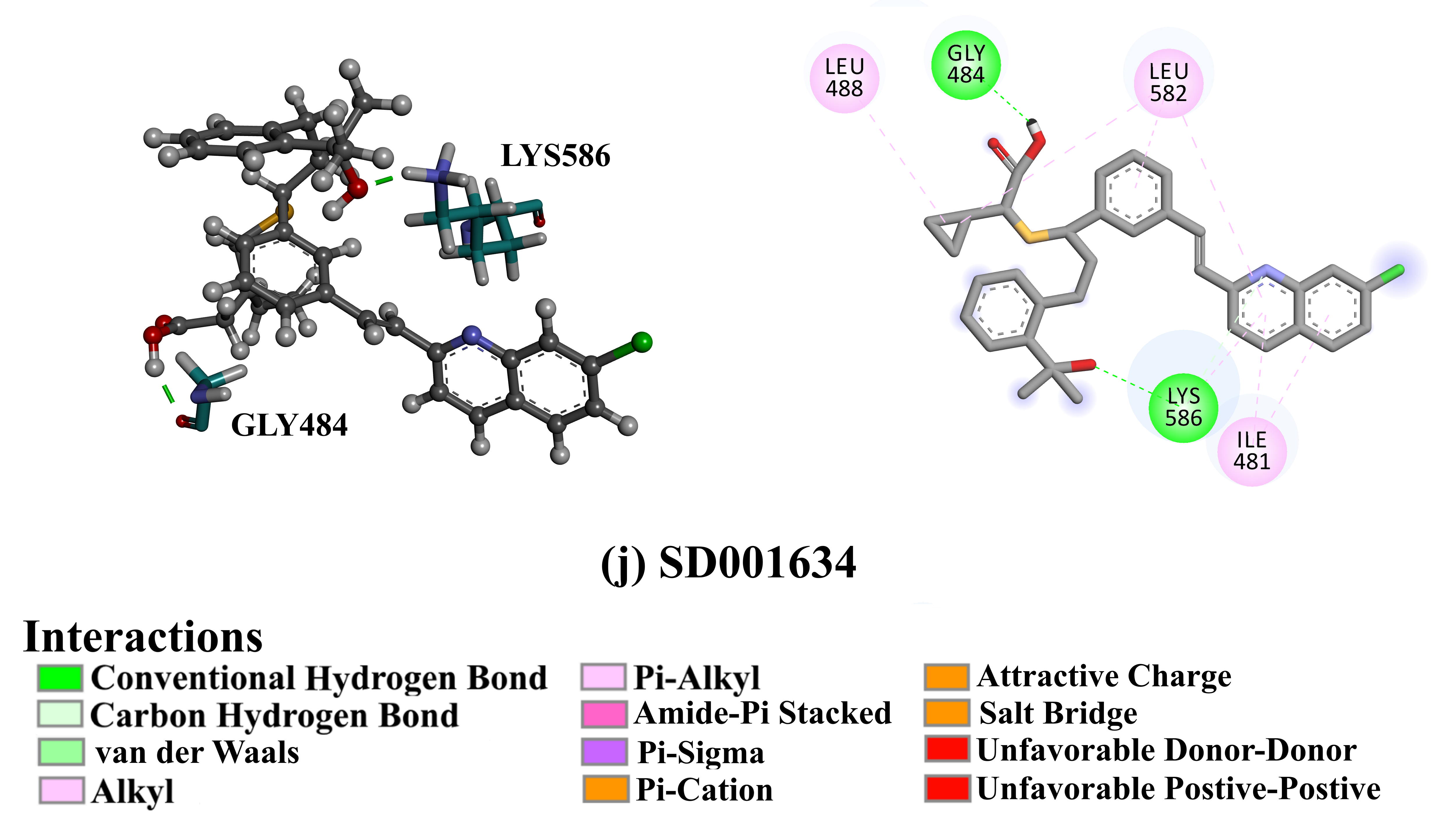
*

**S1 Fig.** *Continued.*
