# Supplementary material for: Repurposing of drug candidates against Epstein–Barr virus: Virtual screening, docking computations, molecular dynamics, and quantum mechanical study
Source: PLoS One. 2024 Nov 15;19(11):e0312100. doi: 10.1371/journal.pone.0312100 (PMC11567563; doi:10.1371/journal.pone.0312100)
Supplement: S2 Table — (DOCX) [file pone.0312100.s003.docx]

**S2 Table.** The anticipated quick and high-accuracy docking scores and MM-GBSA binding energies (in kcal/mol) over 5 ns implicit MDS for the top 125 SuperDRUG2 compounds and KWG towards EBNA1 ^a^.

| No. | **Compound Code** | **Docking Score (kcal/mol)** | | **MM-BSA Binding Energy (kcal/mol)** |
| --- | --- | --- | --- | --- |
|  |  | **Quick** | **High-Accuracy** |  |
|  | **KWG** | **–7.8** | **–7.8** | **–25.8** |
| 1 | SD002534 | –7.9 | –8.2 | –52.4 |
| 2 | SD003350 | –8.9 | –8.9 | –46.0 |
| 3 | SD002868 | –7.8 | –8.0 | –45.2 |
| 4 | SD000750 | –8.0 | –8.6 | –43.8 |
| 5 | SD003513 | –8.6 | –8.7 | –41.9 |
| 6 | SD000463 | –7.8 | –8.1 | –41.1 |
| 7 | SD003857 | –7.8 | –8.0 | –39.1 |
| 8 | SD001047 | –9.3 | –8.6 | –39.0 |
| 9 | SD000076 | –8.4 | –8.7 | –38.8 |
| 10 | SD002195 | –7.9 | –8.1 | –37.8 |
| 11 | SD003940 | –8.7 | –8.8 | –37.6 |
| 12 | SD000308 | –9.5 | –10.1 | –37.4 |
| 13 | SD001650 | –8.3 | –8.3 | –36.7 |
| 14 | SD000472 | –8.5 | –7.9 | –36.4 |
| 15 | SD000062 | –8.8 | –8.8 | –36.2 |
| 16 | SD000345 | –8.3 | –8.3 | –36.2 |
| 17 | SD003067 | –8.4 | –8.7 | –35.9 |
| 18 | SD001170 | –9.1 | –10.0 | –34.9 |
| 19 | SD001159 | –9.6 | –9.9 | –34.7 |
| 20 | SD002855 | –9.2 | –9.7 | –34.5 |
| 21 | SD000839 | –7.9 | –8.0 | –34.4 |
| 22 | SD001644 | –8.4 | –8.4 | –34.1 |
| 23 | SD000705 | –8.3 | –8.3 | –33.9 |
| 24 | SD000932 | –9.0 | –9.7 | –33.3 |
| 25 | SD001263 | –8.4 | –8.4 | –33.1 |
| 26 | SD000691 | –8.2 | –8.4 | –32.1 |
| 27 | SD002322 | –9.0 | –9.6 | –32.0 |
| 28 | SD002561 | –8.8 | –8.9 | –31.9 |
| 29 | SD003303 | –8.0 | –8.4 | –31.9 |
| 30 | SD003840 | –8.0 | –8.2 | –31.7 |
| 31 | SD001955 | –7.9 | –7.9 | –31.4 |
| 32 | SD003398 | –9.0 | –9.0 | –31.0 |
| 33 | SD000670 | –8.9 | –8.9 | –30.5 |
| 34 | SD000787 | –7.9 | –7.9 | –30.4 |
| 35 | SD001634 | –8.0 | –7.9 | –30.4 |
| 36 | SD000609 | –8.2 | –8.2 | –30.3 |
| 37 | SD000944 | –8.0 | –8.1 | –30.0 |
| 38 | SD003363 | –8.2 | –8.5 | –30.0 |
| 39 | SD003725 | –7.8 | –8.4 | –29.9 |
| 40 | SD002572 | –7.9 | –8.4 | –29.9 |
| 41 | SD002109 | –8.2 | –8.2 | –29.8 |
| 42 | SD002439 | –8.2 | –8.3 | –29.4 |
| 43 | SD003355 | –8.0 | –8.0 | –29.3 |
| 44 | SD003858 | –9.5 | –9.5 | –29.3 |

**S2 Table.** *Continued*.

| No. | **Compound Code** | **Docking Score (kcal/mol)** | | **MM-GBSA Binding Energy (kcal/mol)** |
| --- | --- | --- | --- | --- |
|  |  | **Quick** | **High-Accuracy** |  |
| 45 | SD001347 | –7.9 | –7.9 | –28.9 |
| 46 | SD001156 | –9.2 | –9.8 | –28.5 |
| 47 | SD001623 | –8.2 | –8.2 | –28.5 |
| 48 | SD002208 | –7.8 | –7.9 | –28.4 |
| 49 | SD000629 | –8.4 | –8.4 | –28.2 |
| 50 | SD000404 | –8.7 | –9.0 | –28.2 |
| 51 | SD001584 | –9.2 | –9.3 | –28.2 |
| 52 | SD003412 | –8.2 | –8.2 | –27.8 |
| 53 | SD001658 | –8.0 | –8.1 | –27.8 |
| 54 | SD003415 | –9.4 | –9.6 | –27.7 |
| 55 | SD002671 | –8.0 | –8.1 | –27.7 |
| 56 | SD001727 | –8.3 | –8.4 | –27.6 |
| 57 | SD000943 | –8.4 | –8.4 | –27.5 |
| 58 | SD002106 | –8.1 | –8.1 | –27.4 |
| 59 | SD000662 | –7.9 | –8.0 | –27.4 |
| 60 | SD002560 | –8.7 | –8.8 | –27.2 |
| 61 | SD002006 | –9.4 | –9.6 | –27.2 |
| 62 | SD001158 | –8.4 | –9.1 | –27.1 |
| 63 | SD002254 | –8.2 | –8.3 | –27.0 |
| 64 | SD000509 | –8.5 | –8.7 | –27.0 |
| 65 | SD001157 | –8.5 | –9.3 | –26.7 |
| 66 | SD003711 | –8.3 | –8.6 | –26.5 |
| 67 | SD000769 | –8.5 | –8.2 | –26.5 |
| 68 | SD001873 | –7.9 | –8.0 | –26.5 |
| 69 | SD003076 | –7.9 | –7.9 | –26.5 |
| 70 | SD003873 | –8.4 | –8.9 | –26.4 |
| 71 | SD003573 | –8.1 | –8.4 | –26.3 |
| 72 | SD003930 | –8.4 | –8.6 | –26.2 |
| 73 | SD001775 | –7.9 | –8.0 | –26.2 |
| 74 | SD000306 | –8.2 | –8.2 | –26.0 |
| 75 | SD003385 | –8.0 | –8.0 | –25.8 |
| 76 | SD003436 | –8.6 | –8.7 | –25.7 |
| 77 | SD003497 | –9.2 | –9.6 | –25.5 |
| 78 | SD000645 | –8.8 | –8.8 | –25.2 |
| 79 | SD002024 | –8.0 | –8.1 | –24.9 |
| 80 | SD002531 | –7.9 | –8.2 | –24.9 |
| 81 | SD003184 | –8.8 | –9.7 | –24.9 |
| 82 | SD001160 | –9.4 | –9.5 | –24.8 |
| 83 | SD000637 | –8.0 | –8.0 | –24.7 |
| 84 | SD003890 | –8.1 | –8.1 | –24.7 |
| 85 | SD001424 | –8.1 | –8.3 | –24.6 |
| 86 | SD002826 | –8.6 | –8.6 | –24.6 |
| 87 | SD000564 | –8.2 | –8.5 | –24.6 |
| 88 | SD000568 | –8.1 | –8.3 | –24.2 |

**S2 Table.** *Continued*.

| No. | **Compound Code** | **Docking Score (kcal/mol)** | | **MM-GBSA Binding Energy (kcal/mol)** |
| --- | --- | --- | --- | --- |
|  |  | **Quick** | **High-Accuracy** |  |
| 89 | SD001335 | –7.8 | –8.3 | –24.0 |
| 90 | SD002767 | –8.1 | –8.1 | –23.6 |
| 91 | SD003847 | –8.8 | –8.7 | –23.4 |
| 92 | SD003288 | –8.4 | –8.4 | –23.3 |
| 93 | SD001111 | –8.2 | –8.2 | –23.3 |
| 94 | SD002746 | –8.8 | –8.9 | –23.2 |
| 95 | SD003895 | –8.1 | –8.4 | –23.2 |
| 96 | SD000725 | –7.8 | –7.9 | –22.6 |
| 97 | SD000860 | –8.0 | –8.0 | –22.5 |
| 98 | SD000168 | –8.4 | –8.5 | –22.3 |
| 99 | SD000169 | –7.9 | –8.6 | –22.1 |
| 100 | SD003273 | –9.3 | –9.3 | –22.0 |
| 101 | SD001755 | –7.9 | –7.9 | –21.9 |
| 102 | SD000214 | –8.2 | –8.2 | –21.7 |
| 103 | SD000774 | –7.9 | –7.9 | –20.9 |
| 104 | SD002170 | –7.9 | –7.9 | –20.2 |
| 105 | SD001057 | –8.8 | –8.9 | –19.7 |
| 106 | SD006032 | –7.9 | –7.9 | –19.6 |
| 107 | SD000315 | –7.9 | –7.9 | –19.3 |
| 108 | SD001849 | –7.9 | –8.0 | –18.7 |
| 109 | SD003270 | –8.1 | –8.2 | –18.5 |
| 110 | SD000469 | –8.5 | –9.0 | –18.5 |
| 111 | SD000615 | –7.9 | –7.9 | –14.9 |
| 112 | SD001271 | –8.4 | –8.4 | –11.6 |
| 113 | SD002620 | –8.1 | –8.6 | –11.5 |
| 114 | SD000189 | –7.9 | –8.1 | –11.5 |
| 115 | SD000623 | –7.9 | –7.9 | –6.8 |
| 116 | SD003391 | –7.9 | –7.9 | –6.4 |
| 117 | SD003134 | –8.1 | –8.2 | –5.7 |
| 118 | SD000752 | –7.9 | –7.9 | –4.9 |
| 119 | SD003438 | –7.9 | –7.9 | –4.3 |
| 120 | SD001942 | –8.0 | –8.1 | –3.9 |
| 121 | SD000459 | –8.3 | –8.5 | –3.5 |
| 122 | SD000485 | –8.0 | –8.2 | –1.5 |
| 123 | SD003764 | –8.2 | –8.3 | –1.5 |
| 124 | SD000174 | –9.6 | –8.6 | –1.3 |
| 125 | SD003055 | –8.8 | –8.9 | –1.3 |

^a^ Data were arranged according to the 5 ns implicit MM-GBSA binding energy.
