# Supplementary material for: Repurposing of drug candidates against Epstein–Barr virus: Virtual screening, docking computations, molecular dynamics, and quantum mechanical study
Source: PLoS One. 2024 Nov 15;19(11):e0312100. doi: 10.1371/journal.pone.0312100 (PMC11567563; doi:10.1371/journal.pone.0312100)
Supplement: S3 Table — (DOCX) [file pone.0312100.s004.docx]

**S3 Table.** The anticipated quick and high-accuracy docking scores and MM-GBSA binding energies (in kcal/mol) over 5 ns implicit and 5 ns explicit MDS for the top 55 SuperDRUG2 compounds and KWG towards EBNA1 ^a^.

| No. | **Compound Code** | **Docking Score (kcal/mol)** | | **MM-BSA Binding Energy (kcal/mol)** | |
| --- | --- | --- | --- | --- | --- |
|  |  | **Quick** | **High-Accuracy** | Implicit MD | Explicit MD |
|  |  |  |  | 5 ns | 5 ns |
|  | **KWG** | **–7.8** | **–7.8** | **–25.8** | **–33.5** |
| 1 | SD000308 | –9.5 | –10.1 | –37.4 | –46.2 |
| 2 | SD003857 | –7.8 | –8.0 | –39.1 | –43.7 |
| 3 | SD001159 | –9.6 | –9.9 | –34.7 | –42.1 |
| 4 | SD001170 | –9.1 | –10.0 | –34.9 | –41.9 |
| 5 | SD000076 | –8.4 | –8.7 | –38.8 | –41.8 |
| 6 | SD001650 | –8.3 | –8.3 | –36.7 | –41.1 |
| 7 | SD001955 | –7.9 | –7.9 | –31.4 | –39.4 |
| 8 | SD002322 | –9.0 | –9.6 | –32.0 | –39.1 |
| 9 | SD001157 | –8.5 | –9.3 | –26.7 | –38.4 |
| 10 | SD000609 | –8.2 | –8.2 | –30.3 | –36.3 |
| 11 | SD001263 | –8.4 | –8.4 | –33.1 | –36.2 |
| 12 | SD003930 | –8.4 | –8.6 | –26.2 | –36.0 |
| 13 | SD000705 | –8.3 | –8.3 | –33.9 | –35.7 |
| 14 | SD001156 | –9.2 | –9.8 | –28.5 | –35.5 |
| 15 | SD000932 | –9.0 | –9.7 | –33.3 | –35.3 |
| 16 | SD000839 | –7.9 | –8.0 | –34.4 | –34.7 |
| 17 | SD001634 | –8.0 | –7.9 | –30.4 | –34.7 |
| 18 | SD000404 | –8.7 | –9.0 | –28.2 | –34.6 |
| 19 | SD002208 | –7.8 | –7.9 | –28.4 | –33.8 |
| 20 | SD000062 | –8.8 | –8.8 | –36.2 | –32.7 |
| 21 | SD002439 | –8.2 | –8.3 | –29.4 | –32.4 |
| 22 | SD002561 | –8.8 | –8.9 | –31.9 | –32.3 |
| 23 | SD003858 | –9.5 | –9.5 | –29.3 | –32.1 |
| 24 | SD000691 | –8.2 | –8.4 | –32.1 | –30.4 |
| 25 | SD000670 | –8.9 | –8.9 | –30.5 | –30.1 |
| 26 | SD002106 | –8.1 | –8.1 | –27.4 | –29.7 |
| 27 | SD001644 | –8.4 | –8.4 | –34.1 | –29.4 |
| 28 | SD001584 | –9.2 | –9.3 | –28.2 | –29.2 |
| 29 | SD002534 | –7.9 | –8.2 | –52.4 | –28.9 |
| 30 | SD000662 | –7.9 | –8.0 | –27.4 | –28.8 |
| 31 | SD002109 | –8.2 | –8.2 | –29.8 | –28.7 |
| 32 | SD001727 | –8.3 | –8.4 | –27.6 | –28.5 |
| 33 | SD002006 | –9.4 | –9.6 | –27.2 | –28.5 |
| 34 | SD000629 | –8.4 | –8.4 | –28.2 | –27.9 |
| 35 | SD002560 | –8.7 | –8.8 | –27.2 | –27.4 |
| 36 | SD000944 | –8.0 | –8.1 | –30.0 | –27.4 |
| 37 | SD001623 | –8.2 | –8.2 | –28.5 | –27.2 |
| 38 | SD000769 | –8.5 | –8.2 | –26.5 | –27.0 |
| 39 | SD001873 | –7.9 | –8.0 | –26.5 | –26.9 |
| 40 | SD000943 | –8.4 | –8.4 | –27.5 | –26.9 |
| 41 | SD001775 | –7.9 | –8.0 | –26.2 | –26.3 |
| 42 | SD001658 | –8.0 | –8.1 | –27.8 | –26.1 |

**S3 Table.** *Continued*.

| No. | **Compound Code** | **Docking Score (kcal/mol)** | | **MM-GBSA Binding Energy (kcal/mol)** | |
| --- | --- | --- | --- | --- | --- |
|  |  | **Quick** | **High-Accuracy** | Implicit MD | Explicit MD |
|  |  |  |  | 5 ns | 5 ns |
| 43 | SD001158 | –8.4 | –9.1 | –27.1 | –25.5 |
| 44 | SD000306 | –8.2 | –8.2 | –26.0 | –25.0 |
| 45 | SD000472 | –8.5 | –7.9 | –36.4 | –24.0 |
| 46 | SD003711 | –8.3 | –8.6 | –26.5 | –23.9 |
| 47 | SD000463 | –7.8 | –8.1 | –41.1 | –23.1 |
| 48 | SD000509 | –8.5 | –8.7 | –27.0 | –21.9 |
| 49 | SD000787 | –7.9 | –7.9 | –30.4 | –21.5 |
| 50 | SD002254 | –8.2 | –8.3 | –27.0 | –21.1 |
| 51 | SD002195 | –7.9 | –8.1 | –37.8 | –20.9 |
| 52 | SD002868 | –7.8 | –8.0 | –45.2 | –15.8 |
| 53 | SD003840 | –8.0 | –8.2 | –31.7 | –13.1 |
| 54 | SD002671 | –8.0 | –8.1 | –27.7 | –11.7 |
| 55 | SD001347 | –7.9 | –7.9 | –28.9 | –11.6 |

^a^ Data were arranged according to the 5 ns explicit MM-GBSA binding energy.
