# Supplementary material for: Repurposing of drug candidates against Epstein–Barr virus: Virtual screening, docking computations, molecular dynamics, and quantum mechanical study
Source: PLoS One. 2024 Nov 15;19(11):e0312100. doi: 10.1371/journal.pone.0312100 (PMC11567563; doi:10.1371/journal.pone.0312100)
Supplement: S4 Table — (DOCX) [file pone.0312100.s005.docx]

**S4 Table.** The anticipated quick and high-accuracy docking scores and MM-GBSA binding energies (in kcal/mol) over 5 ns implicit and 5 ns and 25 ns explicit MDS for the top 17 SuperDRUG2 compounds and KWG towards EBNA1 ^a^.

| No. | **Compound Code** | **Docking Score (kcal/mol)** | | **MM-BSA Binding Energy (kcal/mol)** | | |
| --- | --- | --- | --- | --- | --- | --- |
|  |  | **Quick** | **High-Accuracy** | Implicit MD | Explicit MD | |
|  |  |  |  | 5 ns | 5 ns | **25 ns** |
|  | **KWG** | **–7.8** | **–7.8** | **–25.8** | **–33.5** | **–34.7** |
| 1 | SD000308 | –9.5 | –10.1 | –37.4 | –46.2 | –44.8 |
| 2 | SD001170 | –9.1 | –10.0 | –34.9 | –41.9 | –44.1 |
| 3 | SD000932 | –9.0 | –9.7 | –33.3 | –35.3 | –42.7 |
| 4 | SD001159 | –9.6 | –9.9 | –34.7 | –42.1 | –42.1 |
| 5 | SD001156 | –9.2 | –9.8 | –28.5 | –35.5 | –41.4 |
| 6 | SD001650 | –8.3 | –8.3 | –36.7 | –41.1 | –40.3 |
| 7 | SD000076 | –8.4 | –8.7 | –38.8 | –41.8 | –39.1 |
| 8 | SD001634 | –8.0 | –7.9 | –30.4 | –34.7 | –36.4 |
| 9 | SD000705 | –8.3 | –8.3 | –33.9 | –35.7 | –36.4 |
| 10 | SD000609 | –8.2 | –8.2 | –30.3 | –36.3 | –34.8 |
| 11 | SD003857 | –7.8 | –8.0 | –39.1 | –43.7 | –34.6 |
| 12 | SD002322 | –9.0 | –9.6 | –32.0 | –39.1 | –34.8 |
| 13 | SD000839 | –7.9 | –8.0 | –34.4 | –34.7 | –34.5 |
| 14 | SD001157 | –8.5 | –9.3 | –26.7 | –38.4 | –34.4 |
| 15 | SD001263 | –8.4 | –8.4 | –33.1 | –36.2 | –34.3 |
| 16 | SD000404 | –8.7 | –9.0 | –28.2 | –34.6 | –33.3 |
| 17 | SD001955 | –7.9 | –7.9 | –31.4 | –39.4 | –32.7 |

^a^ Data were arranged according to the 25 ns explicit MM-GBSA binding energy.
