# Supplementary figures and images for: Repurposing of drug candidates against Epstein–Barr virus: Virtual screening, docking computations, molecular dynamics, and quantum mechanical study
Source: PLoS One. 2024 Nov 15;19(11):e0312100. doi: 10.1371/journal.pone.0312100 (PMC11567563; doi:10.1371/journal.pone.0312100)

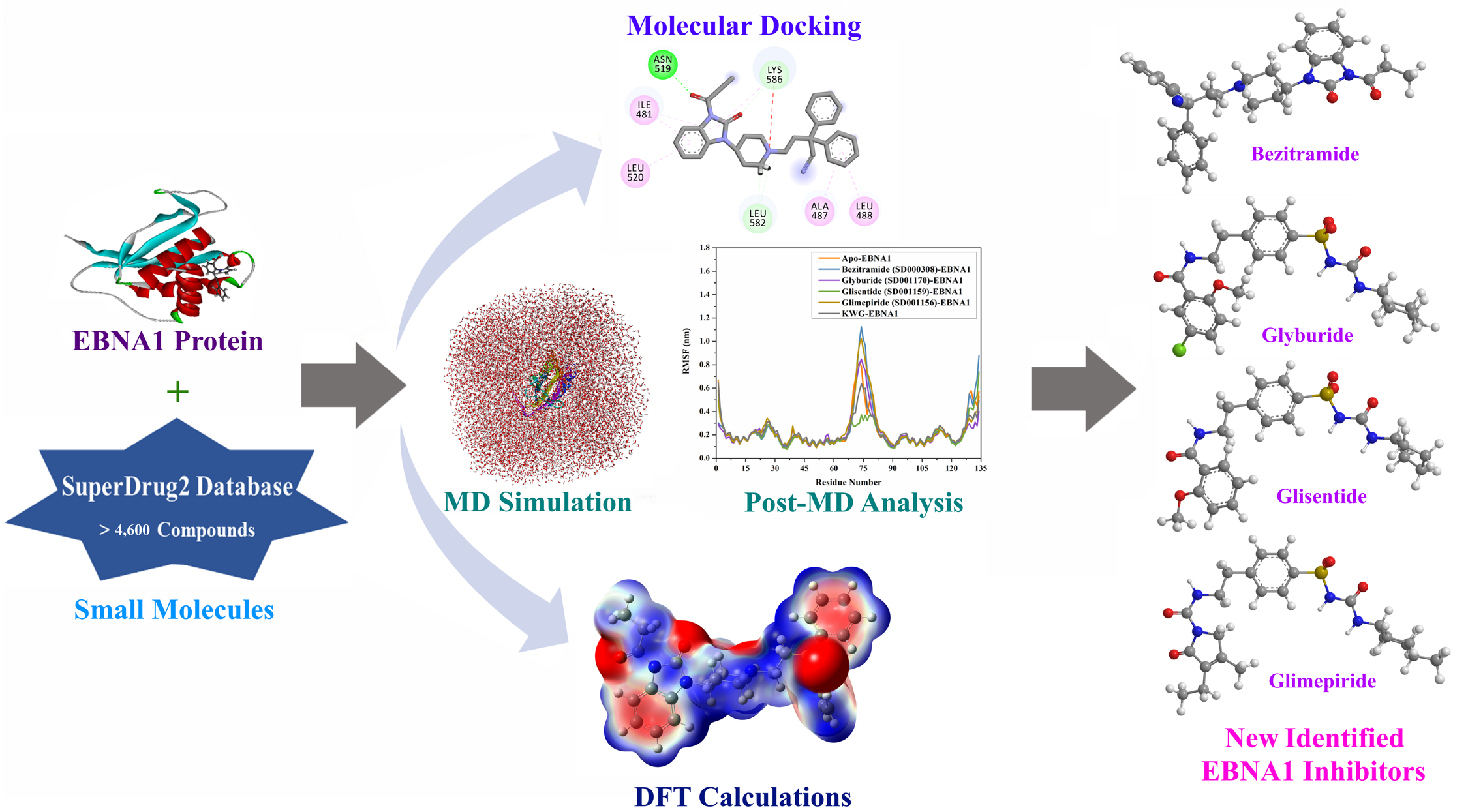

Supplement: S1 Graphical abstract — (TIF) [file pone.0312100.s006.tif]
